# Efficient genome editing in *Pseudomonas syringae* pv. *actinidiae* using the CRISPR/FnCas12a system

**DOI:** 10.1186/s43897-025-00180-0

**Published:** 2025-11-03

**Authors:** Zhenzhen Gou, Yue Wang, Chunyi Qin, Fang Yan, Xiangning Du, Zhengyin Xu, Bo Zhu, Pu Liu, Huanbin Zhou, Gongyou Chen

**Affiliations:** 1 School of Agriculture and Biology/State Key Laboratory of Microbial Metabolism, Shanghai Jiao Tong University, Shanghai, China; 2 Key Laboratory of Urban Agriculture of the Ministry of Agriculture, Shanghai, 200240 China; 3 Anhui Province Key Laboratory of Horticultural Crop Quality Biology, School of Horticulture, Anhui Agricultural University, Hefei, 230036 China; 4 State Key Laboratory for Biology of Plant Diseases and Insect Pests, Institute of Plant Protection, Chinese Academy of Agricultural Sciences, Beijing, 100193 China

**Keywords:** Bacterial canker, Effector, Mutations, Kiwifruit, Virulence

## Abstract

CRISPR-based gene editing has rarely been studied in plant pathogens. In this report, the CRISPR/FnCas12a system was successfully established for gene editing in *Pseudomonas syringae* pv. *actinidiae* (*Psa*), which causes bacterial canker (BC) of kiwifruit. The system was constructed in a *Psa*-suitable vector pBBR1-MCS2 to edit *hopH1* or/and *hopZ5*, which encode effectors only present in *Psa* biovar 3 (*Psa*3) responsible for BC pandemic in kiwifruit worldwide. Two different CRISPR RNAs (crRNA) were designed to edit either *hopH1* or/and *hopZ5*, and two different sets of PCR primers were used to screen deletions of the target genes and the presence of the vector in *Psa*. The deletion in *Psa* was impacted by the position of the DNAs targeted by the crRNAs. The vector-eliminated mutant could receive the editing system iteratively. Interestingly, the double mutant Δ*hopZ5*Δ*hopH1* showed significantly stronger virulence than the wild-type *Psa* on *Actinidia eriantha* cv. White (resistant to BC), but weak virulence on *A. chinensis* cv. Hongyang (highly susceptible to BC), respectively, suggesting that *hopH1* or/and *hopZ5* potentially matches an unknown resistance gene in White. In summary, we have established the CRISPR/FnCas12a genome-editing system to probe gene function in the pathogen and to explore effector-target interactions in kiwifruit*-Psa*-pathosyetem.

**Supplementary Information:**

The online version contains supplementary material available at 10.1186/s43897-025-00180-0.

## Core

A Clustered regularly interspaced short palindromic repeats (CRISPR)-FnCas12a gene-editing system was successfully, efficiently and I teratively conducted to generate hopH1 and hopZ5 mutation in Pseudomonas syringae pv. actinidiae (Psa), which causes bacterial canker of kiwifruit. The deletion mutants were used to determine the role of two effectors in host specificity and virulence. The results show that the established CRISPR/FnCas12a editing system can be used to explore interesting genes in Psa biology, pathogenicity and virulence.

## Gene & Accession Numbers

The genome of M228: CP032631 hopH1: CN228_09540, WP_017704372.1 hopZ5: CN228_09535, WP_020314360.1 AeZLP1: DTZ79_15g09640.1 AcZLP1: Actinidia05846.1.

## Introduction

During growth and development, plants are continually threatened and challenged by pathogens, including viruses, bacteria, and fungi; in response, plants deploy multiple, layered defense mechanisms to thwart pathogen ingress (Ngou et al. 2022). The phytopathogenic bacterium *Pseudomonas syringae* pv. *actinidiae* (*Psa*) causes bacterial canker (BC) of kiwifruit. *Psa* is considered the most destructive pathogen of kiwifruit and causes necrosis and abscission of leaves and buds, shriveling and desiccation of fruits, and dieback of canes and vines (Wang et al. 2019). In 2010, *Psa* infected about 92% of kiwifruit orchards in New Zealand and was added to the European and Mediterranean Plant Protection Organization (EPPO) alert list in 2019. Unfortunately, *Psa* continues to threaten kiwifruit production despite ongoing control efforts (Kim et al. 2020) McCann et al. 2013; 2017). Different kiwifruit cultivars vary in their susceptibility to *Psa*, and breeding for tolerance or resistance to BC remains an important endeavor (Qin et al. 2023).

Numerous methods have been developed for genome editing in bacteria. Zinc finger nuclease (ZFN) technology was an early gene-editing tool that utilizes zinc finger proteins to specifically recognize and cleave DNA sequences (Miller et al. 1985). However, its experimental design complexity and high off-target rates limited its applications (Doyon et al. 2011). Building upon ZFN, scientists developed the more flexible and efficient transcription activator-like effector nuclease (TALEN) technology. TALENs are complexes composed of transcription activator-like effectors and DNA cleavage enzymes, enabling more precise DNA sequence recognition and cutting (Mahfouz et al. 2014) and widely applicable in gene-editing in plants, fish, and human cell (Li et al. 2012; Joung et al. 2013). However, the (TALEN) technology is soon replaced by a groundbreaking clustered regularly interspaced short palindromic repeats (CRISPR)-Cas9 system when Doudna and Charpentier deciphered the mechanism and established it as a universal gene-editing tool (Jinek et al. 2012). This technology was rapidly adopted in bacterial systems (Jiang et al. 2013; Hua et al. 2024). In this system, the CRISPR RNAs (crRNA) assembly interacts with *trans*-activating RNA (tracrRNA) by CRISPR Cas9 to form a single guide RNA (sgRNA), while CRISPR Cas12 can function without tracrRNA (Shmakov et al. 2015). After sgRNA recognizes the target DNA sequence by base pairing, the guide RNA (gRNA) interacts with target DNA, and a R-loop is formed and CRISPR associated proteins (Cas) then act as endonucleases and cleave the DNA fragment (Jinek et al. 2012). In this process, the protospacer adjacent motifs (PAMs) located next to the target sequences are required for recognition of the DNA by sgRNA/Cas protein complex.

CRISPR systems are grouped into Classes 1 and 2 and further subdivided into types I-VI, which vary in the Cas enzymes utilized and editing approaches. Class 1 contains types I, III, and IV, and Class 2 contains types II (Cas9), V (Cas12a), and VI (Cas13a) (Lan et al. 2024). Unlike Class 1, which is comprised of 4–7 Cas protein subunits (Liu et al. 2020), Class 2 enzymes require a single protein and one gRNA to cleave target DNA (Shmakov et al. 2015) and are easier to use than Class 1. Similar to CRISPR/Cas9, CRISPR/FnCas12a (Class 2, type V) is also widely used in gene editing and detection (Kleinstiver et al. 2019) and has a lower off-target rate than CRISPR/Cas9 (Li et al. 2019), Comparatively, the gRNA of CRISPR/Cas9 contains both crRNA and tracrRNA (Jinek et al. 2012),while the gRNA of CasFn12a consists of crRNA alone and can form mature crRNA by self-processing its pre-crRNA (Zetsche et al. 2015). Furthermore, the Cas9 gRNA is usually > 100-bp, whereas the gRNA in CasFn12a requires only 42–44 bp (Zetsche et al. 2015). FnCas12a can cleave DNA strands that are both complementary and non-complementary to crRNA, a process known as *cis* and *trans* cleavage, respectively (Dai et al. 2019). In the CPRISPR/FnCas12a system, a short sgRNA recognizes DNA near the PAM sequence (TTTV) and facilitates cleavage by guiding FnCas12a protein, which results in double-stranded DNA breaks (DSBs) (Li et al. 2018). One mechanism to repair genomic DNA undergoing DSBs is nonhomologous end-joining (NHEJ), where break ends are directly ligated without the need for a homologous template; this process can result in both insertions and deletions in the target DNA (Guo et al. 2018). CRISPR/FnCas12a induced DSBs can be repaired by NHEJ proteins encoding a Ku-like protein (Ku) and an ATP-dependent ligase D (LigD) (Malyarchuk et al. 2007; Lieber. 2010; Yang et al. 2021), but it is not yet known whether the Ku and LigD are required in *Psa* editing.

One traditional strategy to generate mutations in *Psa* is SacB-mediated homologous recombination (Zhang et al. 2022), but this approach is time consuming and not always successful (Schweizer. 1992). Recently, a few reports tried to use CRISPR/Cas editing in phytopathogenic bacteria (Li et al. 2023; Yan et al. 2023). In this study, we successfully use the CRISPR/FnCas12a system in *Psa* to generate deletion mutants in the *hopZ5*/*hopH1* locus of *Psa*. Compared to the SacB-dependent homologous recombination used in our lab (Zou et al. 2011), the CRISPR/FnCas12a technology reduces the time required for constructing edited bacterial strains by nearly half. Our results confirm the utility of CRISPR/FnCas12a in editing the *Psa* genome.

Plants may recognize pathogen-associated molecular patterns (PAMPs) through pattern-recognition receptors (PRRs) on their cell surfaces(Ngou et al. 2024); this can initiate an immune response that is commonly referred to as PAMP-triggered immunity (PTI). As a countermeasure, pathogens can overcome the plant defense response by secreting effector proteins that inhibit PTI. In turn, plants may possess resistance (R) proteins that recognize virulence effectors and elicit effector-triggered immunity (ETI) to inhibit the infection process (Jones et al. 2016). Recognition of effectors via R proteins can result in an immune response that provides a valuable tool in breeding resistant host plants (Lovelace et al. 2023). A critical component of this approach is a thorough analysis of the effectors produced by the pathogen, which often relies on bioinformatics. With respect to *Psa,* at least five biological variants (biovars) have been identified (Qin et al. 2023), and the *Psa*3 is considered the most damaging worldwide (Donati et al. 2020).The five biovars harbor effector proteins that have roles in virulence and host specificity (Scortichini et al. 2012). Regarding the *Psa*3, two effectors, HopH1 and HopZ5, are unique and are co-transcribed (Sawada et al. 2019). HopH1, belonging to XopG/HopH/AvrPtoH family, contains an HEXXH motif, a typical of zinc metallopeptidase, is widely distributed in pathogenic bacteria of animals and plants (Baruch et al. 2011; Wang et al. 2023). The HopH1 knockout strain *Pseudomonas syringae* pv. *tomato* (*Pst* DC3000) showed a significant decrease in virulence on *Arabidopsis* and tomato (Wei et al. 2007). HopZ5, the ​​YopJ family member, exhibits ​​acetyltransferase activity (Jayaraman et al. 2017) and disrupts host cell functions by modifying lysine residues of host proteins (Ma et al. 2015a). In *Arabidopsis*, HopZ5 acetylates the conserved threonine (T166) of RIN4, activating RPM1-dependent immunity. This modification is counteracted by the host deacetylase SOBER1 to fine-tune immune responses (Choi et al. 2021). Besides this, HopZ5 (DC3000) is also recognized by HOPZ-ACTIVATED RESTANCE 1 (ZAR1) in *Arabidopsis* (Bi et al. 2021; Jacob et al. 2021). However, it is still unclear whether these two effectors in *Psa*3 have the similar roles in kiwifruit. Given the fact that *A. chinensis* cv. Hongyang is susceptible to *Psa3* and *A. eriantha* cv. White resistant (Wu et al. 2009), implying that the HopH1 and HopZ5 effectors unique in *Psa*3 may play host specificity, we sought to use the *hopH1* and *hopZ5* genes as the targets for the CRISPR/FnCas12a editing in *Psa*, and Hongyang and White as the hosts for bacterial virulence test when genome-edited mutants of *Psa* are generated.

## Results

### Development of a CRISPR/FnCas12a-based genome editing system in Psa with heterologous NHEJ proteins

**Fig. 1:**
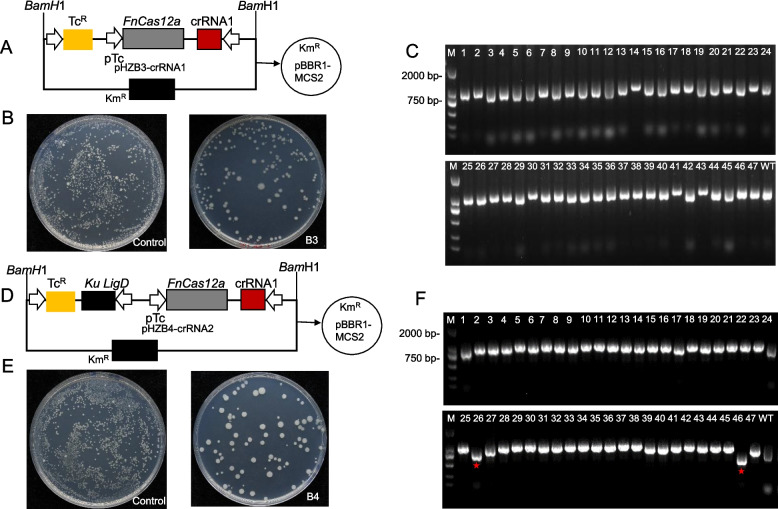
CRISPR/FnCas12a system with heterologous NHEJ proteins. **A** The structure of pBBR-B3-crRNA1. The recombinant plasmid pBBR-B3-crRNA was constructed as follows: pHZB3-crRNA1 was digested with *Bam*HI, and the resulting fragment was ligated into the *Bam*HI-linearized pBBR1-MCS2 vector using T4 DNA ligase. **B** The plasmid pBBR-B3-crRNA1 was conjugated into M228 and grown on antibiotic-containing plates. As a control, an empty vector lacking the crRNA1 was tested under identical conditions. **C** 1–47, PCR analysis was performed on 47 single colonies randomly selected from agar plates; WT, *Psa* M228. **D** The structure of pBBR-B4-crRNA1. The plasmid pHZB4 was upgraded from pHZB3 by incorporating mtKu and mtLigD proteins, The recombinant plasmid pBBR-B4-crRNA was constructed by digesting pHZB4-crRNA1 with *Bam*HI and ligating the resulting fragment into the *Bam*HI-linearized pBBR1-MCS2 vector using T_4_ DNA ligase. **E** The plasmid pBBR-B4-crRNA1 was conjugated into M228 and grown on antibiotic-containing plates. As a control, an empty vector lacking the crRNA1 was tested under identical conditions. **F** 1–47, PCR analysis was performed on 47 single colonies randomly selected from agar plates, Successful knockouts are labeled with red pentagrams; WT, *Psa* M228

The heterologous CRISPR/FnCas12a system mediated by a vector pHM1 is previously used to have genome-editing in plant bacterial pathogens *X. oryzae* pv. *oryzae* PXO99^A^ and *P. syringae* pv. *tomota* DC3000 (Yan et al. 2023), but the system could not be transferred into *Psa* M228 by electrotransformation. To solve this, we turned to use biparental conjugation method to deliver the system into M228 (Laflamme et al. 2020). Since the donor strain *Escherichia coli* (*E. coli*) S17-1 λpir harboring spectinomycin resistance (Sp^R^) (Table S1), a kanamycin-resistant (Km^R^) broad-host-range cloning vector pBBR1-MCS2 was selected and suitable to M228 (Laflamme et al. 2020). Thus, the FnCas12a module with an inducible tetracycline promoter (pTc) to reduce the toxicity of FnCas12a to bacterial cells was then constructed in pBBR1-MSC2 (Fig. 1A). To know whether the Ku and LigD are required in *Psa* editing, two constructs were made. One was pHZB3 which only carried the CRISPR/FnCas12a and the gRNA targeting the virulence gene *hopH1* (Fig. 1A), and another was pHZB4 carrying the Ku and LigD coding genes, the CRISPR/FnCas12a, and the gRNA targeting *hopH1* (Fig. 1D). The two constructs were transferred into the vector pBBR1-MSC2 and resulted in pBBR-B3-crRNA1 and pBBR-B4-crRNA1 (Table S1), respectively. The new generated constructs were then transferred into the S17-1 λpir strain, giving S17-1(pBBR-B3-crRNA1) and S17-1(pBBR-B4-crRNA1), respectively (Table S1). By biparental conjugation, the introduction of the pBBR-B3-crRNA1 into M228 resulted in significantly fewer single colonies growing on the plate compared to the empty vector pBBR-B3 (Table S1) control (Fig. 1B). Using the primer pair of hopH1-F/R (Table S2) to PCR-amplify 47 randomly selected single colonies of the conjugated transformants on the Luria–Bertani (LB) plate complemented with Nalidixic acid (Nal), Cefazoline (Cfz) and kanamycin (Km) antibiotics, the sequencing PCR products showed no successful knockout mutants obtained (Fig. 1C), demonstrating that the cleavage activity of FnCas12a exerts cytotoxic effects on host cells without the Ku and LigD modification. Following the same protocol, the introduction of the pBBR-B4-crRNA1 into M228 produced Nal, Cfz and Km resistant colonies (Fig. 1E) and the PCR product sequencing indicated that there were two knocked-out mutants which showed shorter in the hopH1 gene (Fig. 1F), suggesting that the CRISPR/FnCas12a editing system needs Ku and LigD to repair DSBs. The above results demonstrated that FnCas12a effectively executed its cleavage function, and the NHEJ proteins effectively repairs FnCas12a-induced DSBs in *Psa* (Fig. 1C, 1F).

### Generation of hopH1-deleted mutants with CRISPR/FnCas12a

**Fig. 2:**
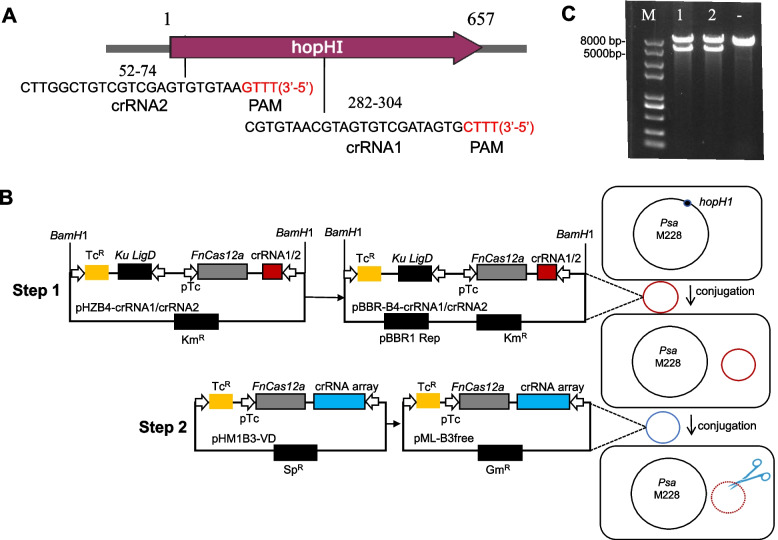
CRISPR/FnCas12a-mediated genome editing in *Psa*. **A** Location of crRNA1 and crRNA2 target regions in *hopH1*; PAM sequences are shown in red. Numbers represent nucleic acid residues. **B** Strategy for *hopH1* editing in *Psa* M228 using the CRISPR/FnCas12a system. In step 1, crRNA1 and crRNA2 were separately inserted into *Sap*I-digested pHZB4, resulting in pHZB4-crRNA1/2, respectively. Next, FnCas12a/crRNA-expression cassettes in pHZB4-crRNA were cloned into pBBR1-MCS2 at the *Bam*HI site, resulting in pBBR-B4-crRNA1/2; these constructs were introduced separately into *Psa* via conjugation. The genomic copy of *hopH1* was edited, resulting in *hopH1* mutants containing pBBR-B4-crRNA1/2 *in trans.* In step 2, pML-B3free was constructed from pHM1B3-VD (Yan et al. 2023); the latter contains an array of two crRNAs that target *oriV* and *LigD* in pBBR-B4-crRNA1/crRNA2*.* The pHM1B3-VD construct was digested with *Bam*HI, and the operon containing *oriV* and *LigD* crRNAs (depicted as a blue rectangle) was integrated into pML123, resulting in pML-B3free. The pML-B3free construct was transferred via conjugation into *Psa* mutants that harbor either pBBR-B4-crRNA1/2. Constructs pBBR-B4-crRNA1/2 each contain a *Ku/LigD* operon that is targeted and cleaved by the crRNA array in pML-B3free (Yan et al. 2023). This eliminates the pBBR-B4-crRNA1/2 plasmids, which results in *Psa* mutants without plasmids*.* pBBR-B4-crRNA1/2 is depicted as red circles, and pML-B3free is depicted as a blue circle. **C** Plasmids were isolated from *E. coli* S17-1 λpir containing pBBR-B4-crRNA1/2 and digested with *Bam*HI. Lanes M, marker; 1, pBBR-B4-crRNA1; 2, pBBR-B4-crRNA2; (-), pBBR1-MCS2

The constructed plasmid pBBR-B4-crRNA (Table S1) was introduced into M228 via biparental conjugation by utilizing the donor strain *E. coli* S17-1 λpir. Thus, the crRNA1 protospacer (23 bp) towards TTTV PAM for *hopH1* targeting were easily cloned, and the resulting FnCas12a/crRNA-expressing cassettes were simply released through *Bam*HI digestion and integrated into pBBR1-MCS2 vector (Fig. 2A). To compare the deletion efficiency in the hopH1 locus, another crRNA2 was designed (Fig. 2A). As the case for crRNA1, the crRNA2 was cloned in *Sap*I-digested pHZB4, resulting in pHZB4-crRNA2 (Table S1) and the resulting FnCas12a/crRNA-expressing cassettes was simply released through digestion and integrated into pBBR1-MSC2 vector (Fig. 2B). pBBR-B4-crRNA1 or pBBR-B4-crRNA2 was transformed into S17-1 λpir via heat shock, followed by Km^R^ selection and PCR screening with primers RV-M/pBBR-B4-check-R (Table S2) to determine whether the vector was present in S17-1 λpir. The plasmids extracted from S17-1 λpir were digested with *Bam*HI indicating that the presence of pBBR-B4-crRNA1 or pBBR-B4-crRNA2(Fig. 2C).

The first step for CRISPR/FnCas12a editing in *Psa* is that the pBBR-B4-crRNA1/2 construct harbored by S17-1 λpir was transferred into M228 by conjugation (Fig. 2B), Expression of FnCas12a in *Psa* was induced in LB containing Anhydrotetracycline (aTc) for 1.5 h, and cells were then plated to LB agar containing Nal, Cfz and Km. Nal and Cfz are to eliminate *E. coli*. Km is to eliminate wild-type *Psa* and indicated that the pBBR-B4-crRNA constructs were retained in the *Psa*. In previous experiments, it was observed that even in the presence of the inducible promoter aTc, the plasmid pBBR-B4-crRNA1 still exhibited self-cleavage in *Psa*. Therefore, to verify the success of targeted-gene knockout, it is essential not only to detect the knockout sequence but also to confirm the presence of the plasmid. We employed two pairs of primers: one pair (CRISPR-check-F/R (Table S2)) to detect the targeted-gene knockout and another (RV-M/pBBR-B4-check-R) to assess plasmid retention.

**Fig. 3:**
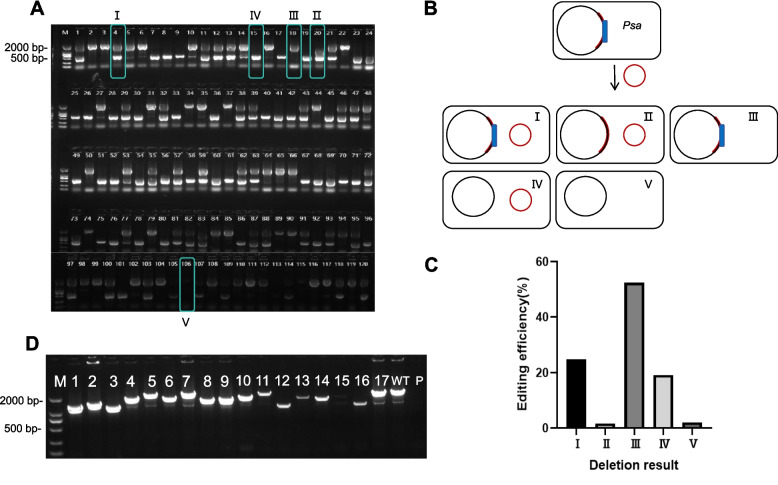
Two primer pairs are needed for efficient detection of CRISPR/FnCas12a-mediated gene editing. **A** Individual *Psa* colonies (*n* = 120) were subjected to PCR with primer pair CRISPR-check-F/R to detect editing in *hopH1.* A PCR product less than 2074-bp indicated that a deletion occurred in the vicinity of *hopH1*. Primers RV-M/pBBR-B4-check-R were used to detect whether pBBR-B4-crRNA1/2 (576-bp) was maintained. Five size categories of PCR products were obtained: I, 2074 and 576-bp; II, < 2074 and 576-bp; III, 2074-bp; IV, 576-bp; and V, 0-bp. **B** Schematic showing the five types of edited products. Genomic DNA and pBBR-B4-crRNA are depicted by the black and red circles, and *hopH1* and flanking sequences are shown in blue and red shading, respectively. **C** Distribution and editing efficiency of the five types of PCR products in the 624 *Psa* colonies. **D** Seventeen type II mutants were subjected to PCR with primer pair CRISPR-check-F/R to detect editing in *hopH1.* A PCR product less than 2074-bp indicated that a deletion occurred in the vicinity of *hopH1*. Lanes: M, marker; 1–17, *Psa* type II mutants; WT, *Psa* M228; P, pBBR-B4-crRNA

To preliminarily assess whether the FnCas12a system successfully knocked out the target sequence, specific primers CRISPR-check-F/R were designed for PCR amplification, where the amplification patterns were analyzed to verify the presence of genomic deletions. In the wild-type *Psa*, amplification with the CRISPR-check-F/R primer pair resulted in a 2074-bp product that contained *hopH1*; In the mutants, the amplification of PCR products less than 2074-bp suggested that a deletion had occurred (Fig. 3A). The absence of PCR products indicated that the sequence targeted by FnCas12a-mediated knockout system were outside the primer binding regions. Initial experiments revealed unstable maintenance of the pBBR-B4-crRNA in host cells, where autonomous cleavage mediated by the CRISPR system likely occurred upon introduction into *Psa*, resulting in failed to knockout. RV-M/pBBR-B4-check-R was used to evaluate whether the pBBR-B4-crRNA1/2 vector was maintained, and the absence of a 576-bp PCR product indicated that the plasmid had undergone self-cleavage (Fig. 3A). A total of 624 single transconjugant colonies were randomly selected and analyzed by PCR with two pairs of primers, and five categories of PCR products were identified (Fig. 3A, B, C). These categories included type I, with 2074 and 576-bp PCR products; II, a product less than 2074-bp and a 576-bp product; III, a 2074-bp product; IV, a 576-bp product; and V, no products (Fig. 3A). In type I, the 2074 and 576-bp PCR products indicated that pBBR-B4-crRNA1/2 was intact and *hopH1* was not deleted. Randomly selected type I, single colonies were subjected to Sanger sequencing. The sequencing results showed no nucleotide deletions, but these data are not presented in the manuscript; 23.56% of *Psa* colonies exhibited this genotype (Fig. 3C). Type II contained PCR products less than 2074-bp and a 576-bp product, which indicated that the target fragment was partially or fully deleted and pBBR-B4-crRNA was intact; 2.72% colonies were type II (Fig. 3C). Type III colonies contained a single 2074-bp product, indicating that *hopH1* was intact and the knockout plasmid underwent self-cleavage; 52.50% colonies were this genotype (Fig. 3C). Type IV colonies exhibited a single 576-bp product; this indicated that pBBR-B4-crRNA1/2 was intact and editing occurred, but the deleted sequence was outside the range detected by the primers. The use of the two primer sets indicated that 19.23% colonies had the type IV genotype (Fig. 3C). In type V, no PCR products were obtained, indicating that the edited sequence was outside the detection range of CRISPR-check-F/CRISPR-check-R and pBBR-B4-crRNA1/2 was no longer present; 2.08% single colonies had this genotype (Fig. 3C).

### Characterization of edited Psa mutants

**Fig. 4:**
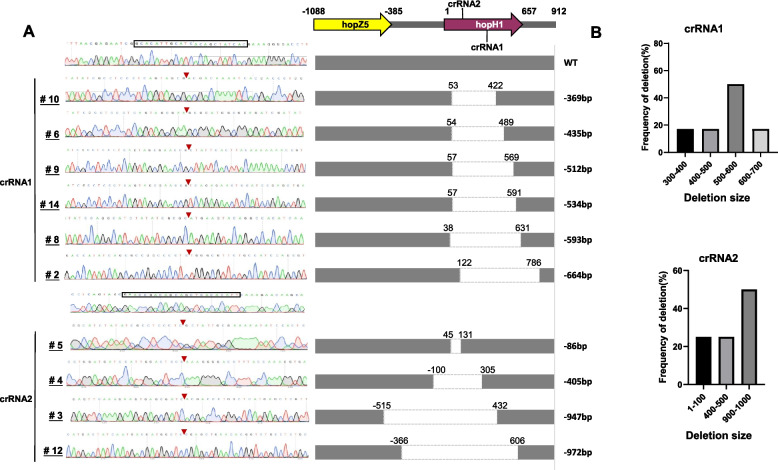
Analysis of 10 *Psa* mutants generated by CRISPR/Cas12a editing. **A** Sequence analysis and functional maps of wild-type *Psa* M228 and 10 *hopH1* deletion mutants generated by crRNA1 (#10, 6, 9, 14, 8, 2) or crRNA2 (#5, 4, 3, 12). The uppermost sequence is wild-type M228; the chromatograms and functional maps represent M228 and 10 independent mutants. The crRNA1 and crRNA2 sequences are shown in rectangles above the chromatograms. The inverted red triangles in the chromatograms indicate the Cas12a cleavage sites. The dashed open rectangles in the functional maps show the extent of deletions in *hopH1*, and deletion sizes are noted on the right. **B** Frequency of distribution vs. deletion size for editing with crRNA1 and crRNA2

Seventeen of the 624 mutant colonies mediated by the two crRNAs were collected for further analysis; these colonies contained a band smaller than 2074-bp and the vector band (576-bp) and were categorized as type II (Fig. 3A, D). These 17 colonies were sequenced, and 10/17 exhibited genome editing with deletions (Fig. 4A). The deletions spanned the PAMs, and there was no tendency for deletions to occur upstream or downstream of the PAMs (Fig. S1). The size of the deletions varied widely; the smallest deletion occurred in mutant #5 (86-bp), and the largest deletion (972-bp) was observed in mutant #12 (Fig. 4A).

The two crRNAs used for editing mapped to different positions with respect to *hopH1*. crRNA1 mapped to the middle of *hopH1* at nucleotide target site (nts) 282–304 (Fig. 2A); six mutants (#10, 6, 9, 14, 8, and 2) were obtained by crRNA1 with deletions ranging from 369–664- bp (Fig. 4A). Mutants #10, 6, 9, 14, and 8 contained deletions within the *hopH1* coding sequence, whereas mutant #2 contained a deletion in *hopH1* and part of the downstream sequence (Fig. 4A). crRNA2 mapped closer to the 5’ end of *hopH1* at nucleotides 52–74 (Fig. 2A). Mutants #5, 4, 3, and 12 were generated with crRNA2 (Fig. 4A); mutant #5 contained a deletion within *hopH1*, mutants #4 and 12 contained deletions in *hopH1* and its upstream, and mutant #3 contained a deletion in *hopH1* that extended the upstream into *hopZ5* (Fig. 4A).

Subsequently, we tested whether additional gene knockouts could be performed in *Psa* strains that had already undergone prior gene deletion. To complement the deletion mutants, the second step was necessary to eliminate the pBBR-B4-crRNA vector. To address this, a crRNA array was utilized that contained crRNA array that target the *oriV* replicon and *LigD*; these crRNA array were present in pHM1B3-VD (Yan et al. 2023). The pHM1B3-VD was digested with *Bam*HI, and the sequence carrying crRNA array was integrated into pML-123, resulting in pML-B3free (Table. S1). The pML-B3free construct was transferred by conjugation into *Psa* mutants #8 to eliminate the pBBR-B4-crRNA1 vector (Step2, Fig. 2B; Fig. S2A) by the following selection: the transconjugants were plated to LB agar containing Nal and Cfz to eliminate S17-1 λpir and screened on LB plates with and without Km. Sensitivity to Km indicated that the plasmid curing was successful and then was verified by colony-PCR with the primers RV-M/pBBR-B4-check-R to display the absence of pBBR-B4-crRNA1 (Fig. S1B). A *hopH1*-deleted and plasmid-absent mutant, hereby as Δ*hopH1*, was further used in this study.

### Generation of hopH1-hopZ5 double mutants in Psa with CRISPR/FnCas12a

**Fig. 5:**
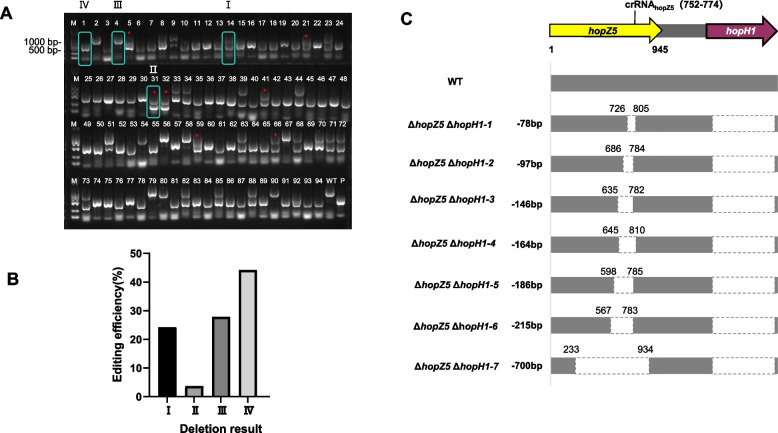
Knock out *hopZ5* in the in the Δ*hopH1* background. **A** Individual *Psa* colonies (*n* = 94) were subjected to PCR with primer pair Check-*hopZ5*-F/Check-*hopZ5*-R to detect editing in *hopZ5.* A PCR product less than 1041-bp indicated that a deletion occurred in *hopZ5*. Primers RV-M/pBBR-B4-CHECK-R were used to detect whether pBBR-B4-crRNA_hopZ5_ (576-bp) was maintained. Four size categories of PCR products were obtained: I, 1041 and 576-bp; II, < 1041 and 576-bp; III, 1041-bp; IV, 576-bp. type II mutants are labeled with red pentagrams.** B** Distribution and editing efficiency of the 4 types of PCR products in the 190 colonies. **C** Sequence analysis and functional maps of wild-type *Psa* M228 and 7 *hopZ5* deletion mutants in the Δ*hopH1* background. The uppermost sequence is the hopZ5 and hopH1 loci, location of crRNA_hopZ5_ targeting in *hopZ5*; the maps represent *Psa* M228 and 7 independent *hopZ5*-deleted mutants in the Δ*hopH1* background. The dashed open rectangles in the functional maps show the extent of deletion sequence, and deletion sizes are noted on the left

Although a double deletion in mutant #3 was realized in *hopH1* and *hopZ5* locus by the crRNA2 with CRISPR/FnCas12a (Fig. 4A), it is unclear whether the second genome-editing event can be occurred in another gene locus when one gene is edited by the same CRISPR/FnCas12a system. For this, a crRNA_hopZ5_ was designed and placed in the construct pBBR-B4-crRNA_hopZ5_ (Table S1). Using the Δ*hopH1* as the recipient to receive pBBR-B4-crRNA_hopZ5_ following the same procedure mentioned above, a total of 190 single colonies from the antibiotic-selective plates were subjected to PCR with the two pairs of primers RV-M/pBBR-B4-check-R and Check-*hopZ5*-F/R (Table S2) for mutant selection. Among the 190 colonies screened, 8 mutants exhibited successful knockout events in *hopZ5* (Fig. 5A). Similar to previous *hopH1* knockout analyses, four distinct amplification patterns (Type I, Type II, Type III and Type IV) were observed, with Type IV notably absent (Fig. 3C, Fig. 5B). The knockout efficiency for *hopZ5* was determined to be 4.21% (Fig. 5B). Subsequent Sanger sequencing validated 7 mutants harboring deletions ranging from 78-bp to 700-bp in the target region (Fig. 5C), indicating that *hopZ5* was successfully deleted in the Δ*hopH1* background. This demonstrates that the CRISPR/Cas12a system can iteratively perform sequential gene knockouts in genetically modified strain after elimination of the pBBR-B4-crRNA1, the pBBR-B4-crRNA_hopZ5_ vector in the mutant Δ*hopZ5*Δ*hopH1-7* was also dispelled by the pML-B3free, resulting in a *hopH1-hopZ5* double mutant Δ*hopZ5*Δ*hopH1* for our further study.

### Virulence analysis of edited mutants

**Fig. 6:**
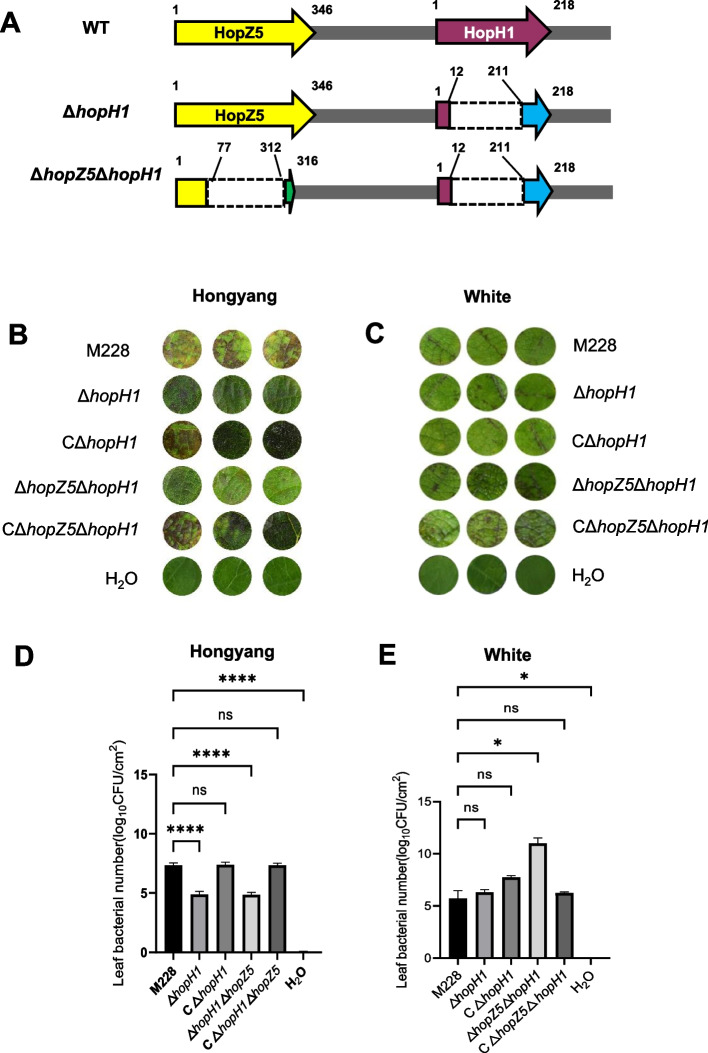
Virulence of edited mutants inoculated to kiwifruit cv. ‘Hongyang’ and cv. ‘White’. **A** Schematic showing location of deletions and mutations in the *hopZ5-hopH1* cluster. Numbers represent amino acids (aa) in the translated protein. The translational product of Δ*hopH1* contained a deletion that spanned 199 aa residues starting from position 12 and ending at residue 211, leading to a frameshift which is depicted with a blue arrow. The Δ*hopZ5*Δ*hopH1* mutant generating an 82 truncated protein in HopZ5, N-terminus region (1–77 aa) of the hopZ5 was remained, the deleted region in *hopZ5* spanned 235 aa from 77^th^ to 312^th^, and the remaining C-terminus was not the real one of HopZ5 because the deletion made a frameshift mutation, which is marked by a green arrow. Dashed lines represent deleted regions. **B** Symptoms on leaf discs of Hongyang that were vacuum-infiltrated with the wild-type *Psa* M228, the *hopH1* mutant (Δ*hopH1*), the *hopH1* complemented strain (CΔ*hopH1*), the *hopZ5-hopH1*mutant (Δ*hopZ5*Δ*hopH1*), the Δ*hopZ5*Δ*hopH1* complemented strain (CΔ*hopZ5*Δ*hopH1*), and water, respectively. **C** Symptoms on leaf discs of White infiltrated with *Psa* M228, Δ*hopH1*, CΔ*hopH1*, Δ*hopZ5*Δ*hopH1*, CΔ*hopZ5*Δ*hopH1*, and water, respectively. Bacterial counts (log_10_ CFU/cm^2^) in inoculated leaf discs of Hongyang (panel **D**) and White (panel **E**). Data were analyzed for significance using GraphPad Prism 9 (https://www.graphpad.com/); each treatment contained three replications, and 15 discs, collected from 4 healthy plants of each cultivar, were used for each replication. three leaf discs were randomly selected from each treatment for symptom record and bacterial growth test. Abbreviations: ns, not significant; *, significant at *P* < 0.05; **, significant at *P* < 0.01

To determine the roles of *hopH1* and *hopZ5* in *Psa* pathogenicity in kiwifruit, two mutants, Δ*hopH1* and Δ*hopZ5*Δ*hopH1* were selected for virulence testing in Hongyang and White. The Δ*hopH1* mutant was derived from the edited mutant #8 and had a 593-bp deletion in the *hopH1* coding region (Fig. 4A). Although the N-terminus of HopH1 was intact, most of amino acids(aa) from 12 to 211 was deleted and a frame shift mutation was identified at the C-terminal end (Fig. 5A). The second edited mutant, Δ*hopZ5*Δ*hopH1*, was derived from Δ*hopZ5*Δ*hopH1-7* and had a 700-bp deletion in *hopZ5*, leading to premature termination in the Δ*hopH1* background (Fig. 5C), The N-terminal region (1–77 aa) of HopZ5 was remained, the 235 aa were truncated from 78 to 312th residue of the innate HopZ5, and the rest C-terminus was not the real one of HopZ5 because the deletion made a frameshift reading (Fig. 6A).

Vacuum infiltration of Hongyang leaf discs with the two mutants resulted in necrotic lesions weaker than to those elicited by the wild-type M228 (Fig. 6B). Analysis of bacterial numbers in the infiltrated leaf discs of Hongyang revealed significant difference between the mutants and wild-type (Fig. 6D). When the complemented strain of Δ*hopH1* with the *hopH1* gene by trans, the necrotic lesions and bacterial growth in Hongyang were recovered to the wild-type M228 (Fig. 6B, 6D). The above results suggest that HopH1 and HopZ5 are required for bacterial full-virulence in BC-susceptible host Hongyang. In parallel, when White was inoculated with the Δ*hopH1* mutant, the symptoms and bacterial population were indistinguishable from the wild-type M228 (Fig. 6C, 6E), but the Δ*hopZ5*Δ*hopH1* mutant induced severe necrosis on White and the mutant population was significantly higher than the wild-type (Fig. 6E). Interestingly, when pBBR-*hopZ5* + *hopH1* was transferred to the Δ*hopZ5*Δ*hopH1* mutant for complementation, the symptoms and population size reverted to wild-type levels (Fig. 6E), implying that there is an unknown R gene in White which may be matched with *Psa* HopZ5.

### HopH1 interacts directly with ZAR1 homologs of kiwifruit

ZAR1-mediated recognition of the DC3000 effector HopZ5 (Zheng et al. 2022) and *Psa* HopZ5 is homologous to DC3000 HopZ5. This led us to assume that the unknown R protein may be an ZAR1-like protein in White triggered by *Psa* HopZ5. Homology searching from available genome data of kiwifruit (https://kiwifruitgenome.org) using nucleotide-binding leucine-rich repeat receptor (NLR) protein ZAR1 in *Arabidopsis thaliana* showed that there are five homologs of ZAR1 in White and six in Hongyang (Fig. S3A). The closest one in White is DTZ79 15g09640 with the 49.29% identities and Actinidia05846 in Hongyang with 49.38% identities to ZAR1, respectively, and named AeZLP1 (ZAR1-like protein) and AcZLP1 correspondingly (Fig. S3). Since HopZ5 induces rapid hypersensitive response (HR)-like cell death (Jayaraman et al. 2017), this precluded reliable assays to investigate the interaction between HopZ5 and AcZLP1/AeZLP1. As HopH1 is required by *Psa* for full virulence in Hongyang (Fig. 6), we turned to perform Luciferase complementation assay (LCA) to see whether HopH1 interacts with AcZLP1/AeZLP1. As shown in Fig. 3SB, HopH1 directly interacted with AcZLP1 and AeZLP1, indicating two possibilities that HopH1 may promote HopZ5 activity in kiwifruit or HopH1 and HopZ5 form a complex to be recognized by an unknown R protein in White.

## Discussion

The pandemic BC caused by *Psa*3 has severely compromised kiwifruit production worldwide (Donati et al. 2020; Vlková-Žlebková et al. 2024). An understanding of effector biology and function is critical for developing kiwifruit cultivars with improved resistance to the pathogen*,* and a genetic approach is routinely used to assess effector function. SacB-mediated mutagenesis has been used for generating deletions and insertions in *Psa* (Zhao et al. 2019; Hemara et al. 2022; Zhang et al. 2022). This approach is based on the lethal activity of levansucrase, the SacB product; unfortunately, this method can result in spontaneous inactivation of SacB during the screening process and can result in false-positive colonies (Ma et al. 2015b; Lazarus et al. 2019). More efficient gene editing methods for *Psa* are urgently needed. The CRISPR/Cas12a system has shown remarkable advantages for gene editing in various organisms and has been used successfully with bacteria (Arroyo-Olarte et al. 2021; Yan et al. 2023). In this study, we show that CRISPR/FnCas12a can be used for gene editing in *Psa* iteratively.

Interestingly, the *Psa* mutants obtained by CRISPR/FnCas12 editing were nucleotide deletions, whereas CRISPR/Cas9-based editing can result in both insertions and deletions (Kosicki et al. 2022). In grapevine, the mutation induced by CRISPR/dCas12a occurred 15–21 nucleotides deletion downstream of the PAMs (Ren et al. 2023). However, in our study, the *Psa* mutants obtained by CRISPR/FnCas12 editing occurred on both sides of the target gene and were located adjacent to the PAMs (Fig. 4A, Fig. 5B); furthermore, there was no tendency to position upstream or downstream of the PAMs (Fig. S1). The bidirectional deletions induced by CRISPR/FnCas12 in *Psa* ranged from 86 to 972-bp, suggesting that the DSBs induced by FnCas12 nuclease are efficiently repaired by the ectopically expressed NHEJ proteins Ku and LigD. Collectively, this study shows that the combined use of the exogenous CRISPR/FnCas12a system and NHEJ proteins enables high-efficiency editing in *Psa*.

Deletions in bacterial genomic DNA (gDNA) are routinely confirmed by PCR and Sanger sequencing, and primers are designed for verifying deletions in the gDNA, not for determining the status of CRISPR/Cas system. Interestingly, we observed that CRISPR/FnCas12a often underwent self-cleavage (Fig. 3A), and both the target DNA and the pBBR-B4-crRNA plasmid experienced splicing. Consequently, we used two primer pairs for PCR, one set of primers was designed to detect deleted gDNA and the second set was used to detect the vector. If pBBR-B4-crRNA was retained, amplification using the RV-M/pBBR-B4-check-R primers revealed a 576-bp PCR product; the absence of the product indicated that the vector had undergone cleavage. The CRISPR-check-F/R primer set amplified a 2074-bp PCR product containing *hopH1* from the wild-type *Psa* M228, and the recovery of 2074-bp products after editing indicated that the *hopH1* deletion was unsuccessful. Deletions in *hopH1* or adjacent DNA resulted in PCR products smaller than 2074-bp. From a total of 624 colonies, 23.56% colonies where pBBR-B4-crRNA was retained and *hopH1* was intact (Fig. 3C). 2.72% colonies had PCR products less than 2074-bp, which indicated a deletion in *hopH1*; these type II colonies retained the pBBR-B4-crRNA plasmid and were further analyzed by Sanger sequencing. 52.50% colonies (type III) contained the unedited 2074-bp PCR product but lacked the pBBR-B4-crRNA plasmid. In type IV colonies, PCR analysis revealed a single 576-bp product; this indicated that the pBBR-B4-crRNA plasmid construct was retained but the deleted sequence was outside the detection range. However, this also demonstrates the capability of CRISPR/Cas12a to perform long-fragment knockout; 19.23% colonies were in this category. In type V colonies, no PCR products were obtained; this indicated that pBBR-B4-crRNA underwent self-cleavage and the presumed *hopH1* deletion was outside the detectable range; 2.08% colonies had this profile (Fig. 3C). To summarize, out of 624 randomly selected colonies, 17 type II colonies were selected for sequencing analysis. Strains with very small deletions or large deletions outside the detection range were not recovered using the two sets of primers (Fig. 3D). To identify small-fragment deletions undetectable via PCR, 10 Type I single colonies were subjected to Sanger sequencing. The sequencing results revealed no evidence of minor deletions in the target region (result didn’t shown).

Our results show that the crRNA sequence impacts the location of deleted nucleotides. The complementary DNA for crRNA1 mapped to nts 282–304 in *hopH1*, and the deletions associated with crRNA were in the vicinity of the targeted DNA sequence (Fig. 4A). Similar results were obtained with crRNA2, which was complementary to nts 52–74 in *hopH1* (Fig. 4A).

Another advantage of the CRISPR/FnCas12 editing in *Psa* is that the genome-edited mutants can be vector-free. In our study, the Δ*hopH1* and the Δ*hopH1*Δ*hopZ5*, derived from mutant #8 and Δ*hopZ5*Δ*hopH1-7*, respectively, in which the CRISPR/FnCas12 vector are eliminated by the pML-B3free, can be used as the recipients to receive new vectors either for further CRISPR/FnCas12 editing (Fig. 5) or gene complementation.

There are obvious differences in the effector proteins produced by different *Psa* biovars (Vlková-Žlebková et al. 2024). *Psa* can be subdivided into at least five biotypes, and *Psa3* is the most prevalent and widespread (Donati et al. 2020; Qin et al. 2023); furthermore, *hopH1* and *hopZ5* are unique to biovar 3 and do not exist in other *Psa* biovars. HopH1 is a homolog of Rip36, a putative Zn-dependent protease effector that induces HR in nonhost plants (Nahar et al. 2021). HopZ5 has acetyltransferase activity and was shown to trigger HR in *Nicotiana benthamian*a and *Arabidopsis*, furthermore, the acetyltransferase activity was required for avirulence. (Choi et al. 2017; Jayaraman et al. 2017) The *hopZ5* and *hopH1* effectors are co-transcribed and were presumably introduced into *Psa*3 by a transposable element (Mccann et al. 2013). In *Psa*3, *hopZ5* and *hopH1* are thought to be redundant and had little effect on virulence when deleted individually (Jayaraman et al. 2023). However, these two effectors increased growth and colonization when introduced individually into *P. syringae* pv. *actinidifoliorum*, a weak pathogen of kiwifruit (Jayaraman et al. 2023).

In our study, there was significant difference in symptoms or bacterial growth when Hongyang was inoculated with the Δ*hopH1* and the Δ*hopZ5*Δ*hopH1* mutants, compared to those by the wild-type M228 (Fig. 5B). This is consistent with a previous report that deleting the *hopZ5-hopH1* cluster from *Psa* M228 reduced virulence on Hongyang (Zhao et al. 2019), suggesting that both HopH1 and HopZ5 are *Psa* virulence factors in kiwifruit. However, in contrast to the results obtained with Hongyang, virulence of the Δ*hopZ5*Δ*hopH1* mutant on cv.White was significantly higher than the wild-type *Psa* M228. This suggests that the *hopZ5-hopH1* region may be recognized by a potentially unknown R protein in White. In support of this, a recent paper by Wang et al. (2020) showed that *A. eriantha* exhibited a high level of resistance to *Psa*3. HopZ5 can be recognized by the coiled-coil NLRs (CNL)—ZAR1(Chen et al. 2021; Zheng et al. 2022). We performed homologous sequence alignment using the aa sequence of ZAR1 and constructed a phylogenetic tree (Fig. S3A). AeZLP1 in White shares a positive of 49.38% (identity 28.98%) with the aa sequence of *Arabidopsis thaliana* ZAR1 (AtZAR1). AcZLP1 in Hongyang does a positive of 49.26% (identity 28.96%). Subsequently, LCA demonstrated that both the AeZLP1 protein and the AcZLP1 interact with the HopH1(Fig. S3B). It is widely accepted that ​​R genes​​ and their cognate ​​effector proteins​​ function in a ​​dominant manner​​. However, this study reveals a more complex scenario: When HopZ5 is present in a ​​cabbage bacterial strain​​ *P. syringae* pv. *maculicola* (*Psm*), the ​​ZAR1​​ resistance gene can recognize it and activate ​​strong disease resistance​​. Conversely, when HopZ5 resides in a ​​tomato bacterial strain​​ *Pst* DC3000, ZAR1-mediated resistance becomes ​​weak. However, another resistance gene, ​​RPM1​​, exhibits the opposite pattern: it confers robust resistance to DC3000 HopZ5 but fails to recognize HopZ5 in *Psm* (Zheng et al. 2022). These findings demonstrate that the ​​recognition and activation mechanisms​​ of plant R genes against pathogen effectors are far more intricate than previously understood. For example, AcGF14C in Hongyang can recognize HopZ5 (Zhou et al. 2024). However, as a highly susceptible variety, Hongyang did not acquire resistance to BC. This raises the question of whether other factors, such as those described in"arms race"-related studies (Ngou et al. 2022), might influence the process of HR triggered by the R gene's recognition of the effector. The loss of resistance in this process may be a key focus for future research. After the R gene recognizes the pathogen's effector, it typically triggers the plant's HR, leading to localized cell death to restrict pathogen spread (Chang et al. 2022). However, during evolution, pathogens and plants engage in an"arms race."Pathogens continuously evolve their effectors to evade or suppress the plant's immune response (Wei et al. 2018). As a result, although AcZLP1 and AeZLP1 in kiwifruit can recognize HopH1, the recognition efficiency might be low, or the signaling pathway could be incomplete or disrupted, failing to effectively trigger HR and leading to the loss of resistance in Hongyang. Another possibility is that HopH1 may form a complex to promote HopZ5 virulence in Hongyang and avirulence in White with a known R gene. This warrants further investigation.

ZAR1, as a canonical CNL, typically utilizes its N-terminal coiled-coil (CC) domain to recognize pathogen effectors, while the nucleotide-binding adaptor shared by Apaf-1, R proteins, and Ced-4 (NB-ARC) domain mediates oligomerization to form a resistosome (Bi et al. 2021). This resistosome anchors to the cell membrane and establishes a calcium ion (Ca^2^⁺) channel (Jacob et al. 2021). Both AcZLP1 and AeZLP1 possess the characteristic CC and NB-ARC domains of CNL-class proteins. Our experiments demonstrate that although AcZLP1/AeZLP1 exhibit binding capability to the effector HopH1 (Fig. S3B), whether their BED/NB-ARC domains enable oligomerization analogous to ZAR1 remains experimentally unverified. Further investigations using structural biology or biochemical assays are required to elucidate their activation mechanisms.

## Materials and methods

### Plant and bacterial strains

*A. chinensis* var. *chinensis* ‘Hongyang’ and *A. eriantha* ‘White’ were the kiwifruit cultivars used in this study. *Nicotiana benthamiana* plants were grown under a 16 h light/8 h dark cycle in a greenhouse at 22 °C for 1 month before being used. *Psa* M228, a virulent biovar 3 strain (Zhao et al. 2019), was isolated from kiwifruit ‘Hongyang’ grown in Meixian, Shaanxi Province, China. The bacterial strains and plasmids used in this study are shown in Table S1.

*Psa* and *E. coli* strains were grown in LB medium at 28℃ and 37℃, respectively. Antibiotics were used at the following final concentrations in µg/ml: spectinomycin (Sp), 100; kanamycin (Km), 25; anhydrotetracycline (aTc), 100; nalidixic acid (Nal), 6; cefazoline (Cfz), 30; and gentamycin (Gm), 30.

### Plasmid construction

The crRNA sequences were designed using https://chopchop.cbu.uib.no/ and were 23-bp in length. The PAM sequence was TTTV and contained a 3-bp adaptor. The designed sequences were synthesized by Qingke Biotechnology Co. (Beijing, China). Complementary oligonucleotides crRNA-F/R were phosphorylated and annealed to generate double-stranded molecules and inserted into *Sap*I-digested pHZB3/pHZB4 (Li et al. 2023; Yan et al. 2023). Next, the pHZB3-crRNA/pHZB4-crRNA were subcloned as *Bam*HI fragments in pBBR1-MCS2; this resulted in pBBR-B3-crRNA/pBBR-B4-crRNA, which were used for genome editing in *Psa* M228.

pHM1B3-VD contains two crRNAs that individually target *oriV* in pML123 and *LigD* in pBBR-B4-crRNA and was obtained from Yan et al. (Li et al. 2023; Yan et al. 2023). pHM1B3-VD was digested with *Bam*HI, and the fragment containing the two crRNAs was integrated into the *Bam*H1 site of pML123, resulting in pML-B3free (Table S1). All constructs were confirmed by Sanger sequencing, which was provided by BioSune Biotechnology (Shanghai, China).

For LCA, the coding sequence (CDS) of AcZLP1(Actindia 05846) and AeZLP1 (DTZ15g09640) were PCR amplified using the cDNAs, which were reversed from the extracted RNAs from Hongyang and White leaves, respectively, by the kit *TransScript*® One-Step gDNA Removal and cDNA Synthesis SuperMix (TransGen Biotech, Beijing, China) with the primers 771-AcZLP1-F/R and 771-AeZLP1-F/R, and cloned into a pCAMBIA-35S-nLuc vector. giving the constructs nLUC-AcZLP1 and nLUC-AeZLP1 (Table S1). The ORF of *hopH1* was PCR-amplified with the primers 771-HopH1-F/R (Table S2) and ligated into pCAMBIA-35S-cLuc vector, resulting in a cLUC-HopH1 construct (Table S1). All the constructs were sequenced by Sanger sequencing (BioSune Biotechnology, Shanghai, China).

### Conjugative transfer

Plasmid constructs were first transferred into *E. coli* S17-1 λpir, and the resulting cells were cultured to an OD_600_ = 1.5. The *Psa* recipient*s* were cultured to an OD_600_ = 1.5. The strains were washed 2–3 times with sterile water and gently mixed in a 1:10 ratio (S17-1 λpir: *Psa*). The mixture was dispensed to a 0.22 μm filter membrane on LB agar and incubated for 12 h at 28 °C. The mixture was resuspended in sterile water and cultured on LB containing the appropriate antibiotics.

### Validation of gene knockouts

The pBBR-B3-crRNA1, pBBR-B4-crRNA1, pBBR-B4-crRNA2 and pBBR-B4-crRNA_hopZ5_ constructs (Table S1) were transferred to *Psa* strains as described above, and transconjugants were recovered on LB agar containing aTc at 28℃ to induce expression of FnCas12a. Cells were washed 2–3 times with sterile water, plated to LB agar containing Km, Nal and Cfz, and incubated at 28℃ for 3–5 d. Single colonies were randomly selected for validation of gene knockouts with two pairs of primers. One pair of primer is CRISPR-check-F/R or Check-*hopZ5*-F/R. The CRISPR-check-F/R was used to detect deletions in *hopH1*, and Check-*hopZ5*-F/R was used to detect deletions in *hopZ5*. Another primer, RV-M/pBBR-B4-check-R, was used to evaluate the presence of the vector. All the PCR products from genome-editing mutants were analyzed by Sanger sequencing.

### Plasmid curing

*Psa* strains carrying the pBBR-B4-crRNA plasmids were cultured in LB containing Km at 28 ℃ to an OD_600_ = 1.5. *E. coli* S17-1 λpir containing pML-B3free was cultivated in LB containing Sp and Gm to OD_600_ = 1.5 at 37 ℃. Next, *Psa* containing pBBR-B4-crRNA cells (2 ml) were combined with 100 µL of *E. coli* S17-1 λpir containing pML-B3free. Mixtures were dispensed to 0.22 filter membranes and incubated at 28℃ for 12 h; cells were then cultivated in LB with aTc for 1.5 h to induce FnCas12a and plated to LB agar with Nal and Cfz to eliminate *E. coli*. Colonies were then screened on LB plates with and without Km; sensitivity to Km indicated that plasmid curing was successful, and this was verified by PCR with the primers RV-M/pBBR-B4-check-R.

### Complementation of edited mutants

Primers *hopH*1-F/*hopH1*-R and hopZ5-F/hopH1-R (Table S2) were used to PCR-amplify *hopH1* (CN228_09540) and *hopZ5* (CN228_09535) *-hopH1* coding sequences using gDNA of *Psa* M228. PCR products were cloned in pBBR1-MCS2 as *Hin*dIII fragments, which resulted in pBBR-*hopH1* and pBBR-*hopZ5* + *hopH1* (Table S1). Both *hopH1* and *hopZ5* + *hopH1* were expressed from the vector *lacZ* promoter with a FLAG tag verified by Western blotting using a FLAG antibody (TransGen Biotech, Beijing, China). After verification, pBBR-*hopH1* and pBBR-*hopZ5* + *hopH1* were introduced into Δ*hopH1* and Δ*hopH1*Δ*hopZ5*, giving CΔ*hopH1 and* CΔ*hopZ5*Δ*hopH1* (Table S1), respectively.

### Inoculation of kiwifruit and statistical analysis

In the bacterial pathogenicity inoculation experiments conducted in this study, young leaves of uniform age were selected from 4 healthy kiwifruit plants. The virulence of *Psa* strains on kiwifruit Hongyang and White was assessed using 1-cm diameter detached leaf discs, avoiding the midrib, which have been used previously to evaluate *Psa* pathogenicity (Zhao et al. 2019). Bacteria were suspended in 40 ml of sterile water (OD_600_ = 0.3), and leaf discs were inoculated by vacuum infiltration at 0.06 kPa for 10 min. The infiltration was repeated three times, and sterile water was included as a control; Inoculated samples were incubated on 0.8% water agar for 5 d in a growth chamber as described previously (Zhang et al. 2022) and subsequently, three discs was randomly selected for photographic documentation. Infiltrated leaf discs were taken from each treatment, macerated, and suspended in 1 ml sterile water; the solution was subjected to tenfold serial dilutions (10^–1^−10^–6^) and then plated to LB agar for enumeration. Data were analyzed for significance using GraphPad Prism 9 (https://www.graphpad.com/); three leaf discs were randomly selected from each treatment for bacterial growth test, each treatment contained three replications, and 15 discs, were used for each replication.

### LCA

*Agrobacterium* strains carrying nLuc and cLuc (Zhou et al. 2018) were mixed with *Agrobacterium* strain expressing the P19 protein at a 1:1:1 ratio in induction buffer, and then co-infiltrated into tobacco leaves. Two days later, the luciferase substrate was injected into the infiltrated areas. The leaves were excised, placed in darkness for 10 min, and fluorescence signals were captured using a CCD camera. In order to avoid the deviation of transfection efficiency, at least four individual *N. benthamiana* plants were used in each analysis. Induction buffer (50 mM MES, 10 mM MgCl₂, 0.2 mM acetosyringone pH 5.6) was prepared fresh before use. Stock solutions of the components were stored at 4 °C.

## Supplementary Information

Supplementary Material 1: Table S1. Bacterial strains and plasmids used in this study.

Supplementary Material 2: Table S2. Primers used in this study.

Supplementary Material 3: Fig. S1. Location of *hopH1* deletions generated by CRISPR/FnCas12a-mediated gene editing with crRNA1 and crRNA2. The first downstream base adjacent to the PAM is referred to as position 1, and the first upstream base adjacent to the PAM sequence is -1. The *x*-axes show the mutant designations, and the *y*-axes show the extent of deletion events.

Supplementary Material 4: Fig. S2. Plasmid curing and strain complementation. A Schematic showing how the FnCas12a nuclease-mediated cleavages of pML-B3-free that are mediated by *oriV* and *LigD* result in editing and removal of pBBR-B4-crRNA constructs. The red and blue circles represent pBBR-B4 and pML-B3, respectively. B *Psa* mutant #8 contains a 593-bp deletion in *hopH1* and was selected for plasmid curing. pML-B3free was transferred into mutant #8 by conjugation, and colonies were then screened on LB plates with and without Km. Sensitivity to Km indicated that plasmid curing was successful, and this was verified by PCR. Individual *Psa* colonies (*n* = 16) were subjected to PCR with the primers RV-M/pBBR-B4-CHECK-R to detect whether pBBR-B4-crRNA (576 bp) was eliminated. Lanes: M, molecular weight marker; 1–20, single colonies randomly selected after plasmid curing; WT, wild-type *Psa* M228; P, pBBR-B4-crRNA1; -, ddH_2_O.

Supplementary Material 5: Fig. S3. HopH1 interacted with ZAR1 homologs from *Actinidia* species. A The phylogenetic tree of *Arabidopsis* ZAR1 in *Actinidia* species was construuted based on full length protein sequence by the maximum likehood method. B LCA showing that HopH1 interacted with the AeZLP1 and AcZLP1 *in N. benthamiana*. Empty vectors were used as negative controls. Four individual *N. benthamiana* plants were used in each replication, each treatment contained three replications and one similar image was used from three replications.

## Data Availability

All relevant data supporting the findings of this study are available within the paper. The constructs, excluding those which are given by references, used in this study are available from the corresponding author upon request.

## Abbreviations

Aa: Amino acid
AtZAR1: Arabidopsis thaliana ZAR1
AcZLP1: *Actinidia chinensis* ZAR1-like protein
AeZLP1: *Actinidia eriantha* ZAR1-like protein
aTc: Anhydrotetracycline
BC: Bacterial canker
CC: Coiled-coil
CDS: Coding sequence
CNL: Coiled-coil NLRs
CRISPR: Clustered regularly interspaced short palindromic repeats
Cas: CRISPR associated proteins
Cfz: Cefazoline
crRNA: CRISPR RNAs
DSBs: Double-stranded DNA breaks
ETI: Effector-triggered immunity
*E. coli*: *Escherichia coli*
HR: Hypersensitive response
NB-ARC: Nucleotide-Binding adaptor shared by Apaf-1, R proteins, and Ced-4
Gm: Gentamycin
gDNA: Genomic DNA
gRNA: Guide RNA
Km: Kanamycin
Km^R^: Kanamycin-resistance
Ku: Ku-like protein
LB: Luria-Bertani
LCA: Luciferase complementation Assay
LigD: Ligase D
Nal: Nalidixic acid
NHEJ: Nonhomologous end-joining
NLR: Nucleotide-binding Leucine-rich repeat Receptor
Nts: Nucleotide target site
PAMs: Protospacer adjacent motifs
PAMPs: Pathogen-associated molecular
PRRs: Pattern-recognition receptors
*Psm*: *P. syringae* pv. *maculicola*
*Psa*: *Pseudomonas syringae* pv. *actinidiae*
*Psa*3: *Psa* Biovar 3
*Pst*: *Pseudomonas syringae* pv. *Tomato*
pTc: TetR promoter
PTI: PAMP-triggered immunity
*R* proteins: Resistance proteins
sgRNA: Single guide RNA
Sp: Spectinomycin
Sp^R^: Spectinomycin resistance
TALEN: Transcription activator-like effector nuclease
tracrRNA: *trans*-Activating RNA
ZAR1: HOPZ-ACTIVATED RESTANCE 1
ZFN: Zinc finger nuclease
ZLP1: ZAR1-like protein
