# Correction: Efficient genome editing in *Pseudomonas syringae* pv. *actinidiae* using the CRISPR/FnCas12a system

**DOI:** 10.1186/s43897-025-00227-2

**Published:** 2025-12-16

**Authors:** Zhenzhen Gou, Yue Wang, Chunyi Qin, Fang Yan, Xiangning Du, Zhengyin Xu, Bo Zhu, Pu Liu, Huanbin Zhou, Gongyou Chen

**Affiliations:** 1 School of Agriculture and Biology/State Key Laboratory of Microbial Metabolism, Shanghai Jiao Tong University, Shanghai, China; 2 Key Laboratory of Urban Agriculture of the Ministry of Agriculture, Shanghai, 200240 China; 3 Anhui Province Key Laboratory of Horticultural Crop Quality Biology, School of Horticulture, Anhui Agricultural University, Hefei, 230036 China; 4 State Key Laboratory for Biology of Plant Diseases and Insect Pests, Institute of Plant Protection, Chinese Academy of Agricultural Sciences, Beijing, 100193 China

**Correction: Mol Horticulture 5, 60 (2025)**

**https://doi.org/10.1186/s43897-025–00180-0**

Following the publication of the original article (Gou et al. 2025), it is reported that the middle leaf disc indicating H_2_O treatment for Hongyang cultivar at the bottom of Fig. 6B was mistakenly duplicated in Fig. 6C to also display H_2_O treatment for White cultivar due to figure preparation careless.

The incorrect Fig. 6 is:

**Fig. 6:**
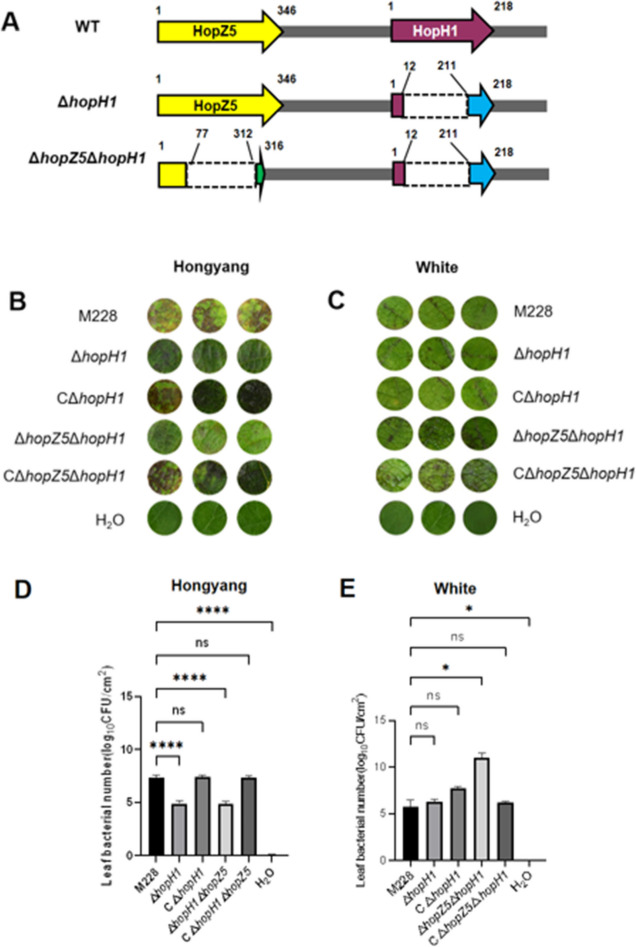
Virulence of edited mutants inoculated to kiwifruit cv. ‘Hongyang’ and cv. ‘White’. **A** Schematic showing location of deletions and mutations in the *hopZ5-hopH1* cluster. Numbers represent amino acids (aa) in the translated protein. The translational product of Δ*hopH1* contained a deletion that spanned 199 aa residues starting from position 12 and ending at residue 211, leading to a frameshift which is depicted with a blue arrow. The Δ*hopZ5*Δ*hopH1* mutant generating an 82 truncated protein in HopZ5, N-terminus region (1–77 aa) of the hopZ5 was remained, the deleted region in *hopZ5* spanned 235 aa from 77 to 312th, and the remaining C-terminus was not the real one of HopZ5 because the deletion made a frameshift mutation, which is marked by a green arrow. Dashed lines represent deleted regions.** B** Symptoms on leaf discs of Hongyang that were vacuum-infiltrated with the wild-type *Psa* M228, the *hopH1* mutant (Δ*hopH1*), the *hopH1* complemented strain (CΔ*hopH1*), the *hopZ5-hopH1*mutant (Δ*hopZ5*Δ*hopH1*), the Δ*hopZ5*Δ*hopH1* complemented strain (CΔ*hopZ5*Δ*hopH1*), and water, respectively. **C** Symptoms on leaf discs of White infiltrated with *Psa* M228, Δ*hopH1*, CΔ*hopH1*, Δ*hopZ5*Δ*hopH1*, CΔ*hopZ5*Δ*hopH1*, and water, respectively. Bacterial counts (log_10_ CFU/cm^2^) in inoculated leaf discs of Hongyang (panel **D**) and White (panel **E**). Data were analyzed for significance using GraphPad Prism 9 (https://www.graphpad.com/); each treatment contained three replications, and 15 discs, collected from 4 healthy plants of each cultivar, were used for each replication. three leaf discs were randomly selected from each treatment for symptom record and bacterial growth test. Abbreviations: ns, not significant; *, significant at *P* < 0.05; **, significant at *P* < 0.01

The correct Fig. 6 is:

**Fig. 6:**
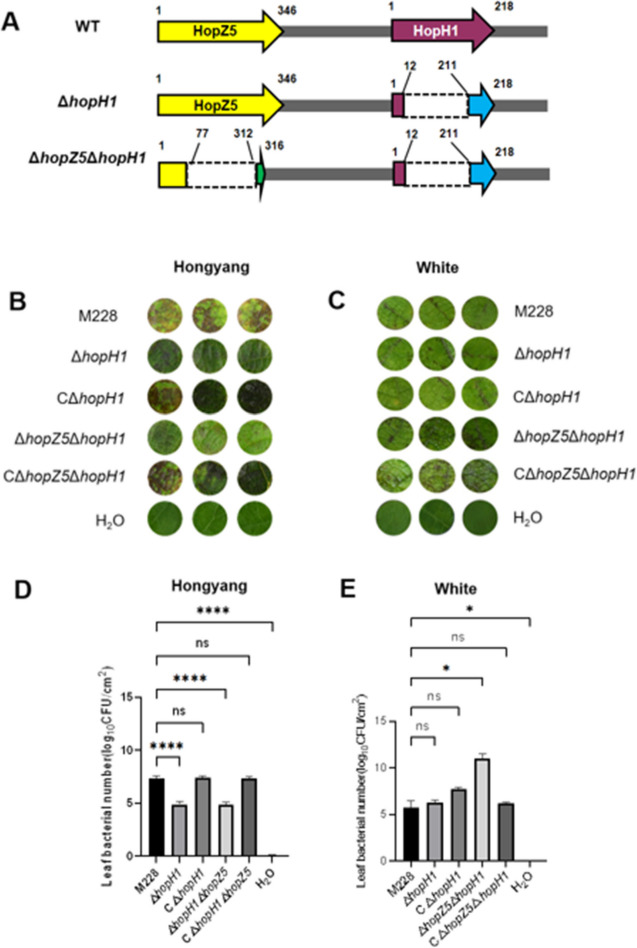
Virulence of edited mutants inoculated to kiwifruit cv. ‘Hongyang’ and cv. ‘White’. **A** Schematic showing location of deletions and mutations in the *hopZ5-hopH1* cluster. Numbers represent amino acids (aa) in the translated protein. The translational product of Δ*hopH1* contained a deletion that spanned 199 aa residues starting from position 12 and ending at residue 211, leading to a frameshift which is depicted with a blue arrow. The Δ*hopZ5*Δ*hopH1* mutant generating an 82 truncated protein in HopZ5, N-terminus region (1–77 aa) of the hopZ5 was remained, the deleted region in *hopZ5* spanned 235 aa from 77 to 312th, and the remaining C-terminus was not the real one of HopZ5 because the deletion made a frameshift mutation, which is marked by a green arrow. Dashed lines represent deleted regions.** B** Symptoms on leaf discs of Hongyang that were vacuum-infiltrated with the wild-type *Psa* M228, the *hopH1* mutant (Δ*hopH1*), the *hopH1* complemented strain (CΔ*hopH1*), the *hopZ5-hopH1*mutant (Δ*hopZ5*Δ*hopH1*), the Δ*hopZ5*Δ*hopH1* complemented strain (CΔ*hopZ5*Δ*hopH1*), and water, respectively. **C** Symptoms on leaf discs of White infiltrated with *Psa* M228, Δ*hopH1*, CΔ*hopH1*, Δ*hopZ5*Δ*hopH1*, CΔ*hopZ5*Δ*hopH1*, and water, respectively. Bacterial counts (log_10_ CFU/cm^2^) in inoculated leaf discs of Hongyang (panel **D**) and White (panel **E**). Data were analyzed for significance using GraphPad Prism 9 (https://www.graphpad.com/); each treatment contained three replications, and 15 discs, collected from 4 healthy plants of each cultivar, were used for each replication. three leaf discs were randomly selected from each treatment for symptom record and bacterial growth test. Abbreviations: ns, not significant; *, significant at *P* < 0.05; **, significant at *P* < 0.01

The original article (Gou et al. 2025) has been corrected.
