# Supplementary material for: Efficient genome editing in Pseudomonas syringae pv. actinidiae using the CRISPR/FnCas12a system
Source: Mol Hortic. 2025 Nov 3;5:60. doi: 10.1186/s43897-025-00180-0 (PMC12581509; doi:10.1186/s43897-025-00180-0)
Supplement: Supplementary file 1 — Supplementary Material 1: Table S1. Bacterial strains and plasmids used in this study. [file 43897_2025_180_MOESM1_ESM.docx]

| **Table S1** Bacterial strains and plasmids used in this study | | | |  |
| --- | --- | --- | --- | --- |
| **Strains** | | **Description** | **Source** | |
| *Escherichia coli* | |  |  | |
| DH5α | | F^-^ *Ф80(lacZ)ΔM15 Δ(lacZYA-argF)U169 deoR endA1 recA1 hsdR17(r*_k_*^-^, m*_k_*^+^) supE44λ^-^thi-1gyrA96 relA1 phoA* | TaKaRa | |
| S17-1 λpir | | Assists in conjugal transfer of plasmid DNA, Sp^R^ | This lab | |
| S17-1(pBBR-B3) | | pBBR-B3 in S17-1 λpir | This study | |
| S17-1(pBBR-B4) | | pBBR-B4 in S17-1 λpir | This study | |
| S17-1(pBBR-B3-crRNA1) | | pBBR-B3-crRNA1 in S17-1 λpir | This study | |
| S17-1(pBBR-B4-crRNA1) | | pBBR-B4-crRNA1 in S17-1 λpir | This study | |
| S17-1(pBBR-B4-crRNA2) | | pBBR-B4-crRNA2 in S17-1 λpir | This study | |
| S17-1(pBBR-B4-crRNA_hopZ5_) | | pBBR-B4-crRNA _hopZ5_ in S17-1 λpir | This study | |
| S17-1(pML-B3free) | | pML-B3free in S17-1 λ pir | This study | |
| *P. syringae* pv. *actinidiae* | | |  | |
| M228 | | Isolated from *A. chinensis* cv. Hongyang, wild-type | (Zhao et al. 2019) | |
| Δ*hopH1* | | *hopH1* deletion mutant #8 of *Psa* M228; lacks 593 bp of the *hopH1* coding region | This study | |
| Δ*hopZ5*Δ*hopH1* | | *hopZ5/hopH1* deletion mutant #3 of *Psa* M228; lacks 947 bp from the 3’ end of *hopZ5* to *hopH1* | This study | |
| CΔ*hopH1* | | Δ*hopH1* deletion mutant containing pBBR-*hopH1 in trans*, Km^R^ | This study | |
| CΔ*hopZ5*Δ*hopH1* | | Δ*hopZ5*Δ*hopH1/* deletion mutant of *Psa* M228 containing pBBR-*hopZ5*+*hopH1 in trans*, Km^R^ | This study | |
| *Agrobacterium tumefaciens* | |  |  | |
| GV3101 | | T-DNA delivery and compatibility with binary vector systems, Rif^R^ | This lab | |
| GV3101(cLuc) | | pCAMBIA-35S-cLuc in GV3101 | This lab | |
| GV3101(nLuc) | | pCAMBIA-35S-nLuc in GV3101 | This lab | |
| GV3101(cLUC-HopH1) | | cLUC-hopH1 in GV3101 | This study | |
| GV3101(nLUC-AcZLP1) | | nLUC-AcZLP in GV3101 | This study | |
| GV3101(nLUC-AeZLP1) | | nLUC-AeZLP in GV3101 | This study | |
| **Plasmids** |  | |  | |
| pHZB3 | Contains FnCas12a/crRNAs under control of a TetR promoter to facilitate plasmid curing, Km^R^ Tet^R^ | | (Yan et al. 2023) | |
| pHZB4 | Contains FnCas12a/crRNAs under control of a TetR promoter and *Ku* and *LigD* to facilitate plasmid curing, Km^R^ Tet^R^ | | (Yan et al. 2023) | |
| pML123 | 11.45-kb broad host range vector, Gm^R^ | | (Labes et al. 1990) | |
| pHM1B3-VD | Contains a crRNA array with two crRNAs that target *oriV* and *LigD* in pHM1 and pBBR1-MCS2; used for eliminating plasmid DNA, Sp^R^ | | (Yan et al. 2023) | |
| pML-B3free | Contains a crRNA array with two crRNAs that target *oriV* in pHM1 and *LigD* in pBBR1-MCS2; used for plasmid curing in *Psa*, Gm^R^ | | This study | |
| pHZB3-crRNA1 | pHZB3 with the crRNA1 array that targets nucleotides 282-304 in *hopH1*and contains the 4-bp PAM sequence, Km^R^ Tet^R^ | | This study | |
| pHZB4-crRNA1 | pHZB4 with the crRNA1 array that targets nucleotides 282-304 in *hopH1*and contains the 4-bp PAM sequence, Km^R^ Tet^R^ | | This study | |
| pHZB4-crRNA2 | pHZB4 with the crRNA2 array that targets nucleotides 52-74 in *hopH1* and contains the 4 bp PAM sequence, Km^R^ Tet^R^ | | This study | |
| pHZB4-crRNA _hopZ5_ | pHZB4 with the crRNA_hopZ5_ array that targets nucleotides 752-774 in *hopZ5* and contains the 4 bp PAM sequence, Km^R^ Tet^R^ | | This study | |
| pBBR1-MCS2 | 5.14-kb broad-host range cloning vector, Km^R^ | | This lab | |
| pBBR-B3 | pBBR1-MCS2 containing FnCas12a, Km^R^ Tet^R^ | | This study | |
| pBBR-B3-crRNA1 | pBBR1-MCS2 containing FnCas12a, and the crRNA1 expression cassette for deleting *hopH1* in *Psa* M228, Km^R^ Tet^R^ | | This study | |
| pBBR-B4 | pBBR1-MCS2 containing *Ku-LigD* and FnCas12a expression cassette, Km^R^ Tet^R^ | | This study | |
| pBBR-B4-crRNA1 | pBBR1-MCS2 containing *Ku-LigD*, FnCas12a, and the crRNA1 expression cassette for deleting *hopH1* in *Psa* M228, Km^R^ Tet^R^ | | This study | |
| pBBR-B4-crRNA2 | pBBR1-MCS2 containing *Ku-LigD*, FnCas12a, and the crRNA2 expression cassette for deleting *hopH1* in *Psa* M228, Km^R^ Tet^R^ | | This study | |
| pBBR-B4-crRNA_hopZ5_ | pBBR1-MCS2 containing *Ku-LigD*, FnCas12a, and the crRNA _hopZ5_ expression cassette for deleting *hopZ5* in *Psa* M228, Km^R^ Tet^R^ | | This study | |
| pBBR-*hopH1* | pBBR1-MCS2 expressing *hopH1* under the control of the vector *lacZ* promoter; contains a FLAG tag, Km^R^ | | This study | |
| pBBR-*hopZ5*+*hopH1* | pBBR1-MCS2 expressing *hopZ5* and *hopH1* under control of the vector *lacZ* promoter with a C-terminal FLAG tag, Km^R^ | | This study | |
| cLuc | pCAMBIA-35S-cLuc | | This lab | |
| nLuc | pCAMBIA-35S-nLuc | | This lab | |
| cLUC-HopH1 | HoH1 is fused with C terminal of Luc | | This study | |
| nLUC-AcZLP1 | AcZLP is fused with N terminal of Luc | | This study | |
| nLUC-AeZLP1 | AeZLP is fused with N terminal of Luc | | This study | |

Labes M, Pühler A, Simon R. A new family of RSF1010-derived expression and lac-fusion broad-host-range vectors for Gram-negative bacteria. Gene. 1990;1:37-46.

Yan F, Wang J, Zhang S, Lu Z, Li S, Ji Z, Song C, Chen G, Xu J, Feng J, Zhou X, Zhou H. CRISPR/FnCas12a-mediated efficient multiplex and iterative genome editing in bacterial plant pathogens without donor DNA templates. PLOS Pathogens. 2023;1:e1010961.

Zhao Z, Chen J, Gao X, Zhang D, Zhang J, Wen J, Qin H, Guo M, Huang L. Comparative genomics reveal pathogenicity-related loci in *Pseudomonas syringae* pv. *actinidiae* biovar 3. Mol. Plant Pathol. 2019;7:923-42.
