# Supplementary material for: Efficient genome editing in Pseudomonas syringae pv. actinidiae using the CRISPR/FnCas12a system
Source: Mol Hortic. 2025 Nov 3;5:60. doi: 10.1186/s43897-025-00180-0 (PMC12581509; doi:10.1186/s43897-025-00180-0)
Supplement: Supplementary file 2 — Supplementary Material 2: Table S2. Primers used in this study. [file 43897_2025_180_MOESM2_ESM.zip › Table S2.docx]

Table S2. Primers used in this study

| Primer name | Sequence (5’-3’) | Description |
| --- | --- | --- |
| crRNA1-F | GATGTGATAGCTGTGATGCAATGTGC | Targeting nts 52-74 in *hopH1* |
| crRNA1-R | AAAGCACATTGCATCACAGCTATCAC |  |
| crRNA2-F | GATAATGTGTGAGCTGCTGTCGGTTC | Targeting nts 282-304 in *hopH1* |
| crRNA2-R | AAAGAACCGACAGCAGCTCACACATT |  |
| crRNA_hopZ5_-F | GATAACCTAGAGAGCATAAGTCCGAG | Targeting nts 752-774 in *hopZ5* |
| crRNA_hopZ5_-R | AAACTCGGACTTATGCTCTCTAGGTT |  |
| FnCas12a-R | GGCACCGAGCTGGATTACCT | Detecting cRNA1 and crRNA2 in pHZB4-crRNA1 and pHZB4-crRNA2 |
| M13-47 | CGCCAGGGTTTTCCCAGTCACGAC | Detecting pBBR-*hopH1* and pBBR-*hopZ5*+*hopH1* together with *hopH1*-F |
| RV-M  pBBR-B4-check-R | GAGCGGATAACAATTTCACACAGG  ATCAAGAACAACCAGGAAGGCAA | Detecting the presence of pBBR-B4-crRNA1 or pBBR-B4-crRNA2 |
| CRISPR-check-F | ATGCTGGCGGTCAGTTGTG | Detecting deletion in *hopH1* |
| CRISPR-check-R | GTGTGTTGGCTGCGATTTTAA |  |
| Check-*hopZ5*-F | ATGGGACTTTGTGCATCAAAACC | Detecting the deletion in *hopZ5* |
| Check-*hopZ5*-R | TTAGGATTCTATCGCTTTTCTTATTTTT |  |
| *hopH1*-F | CGGAAGCTTAATGATCACTCCATCTCGATATCCAGGC | For cloning *hopH1; Hin*dIII and *Eco*RI sites are underscored |
| *hopH1*-R | CCGGAATTCCTACTTATCGTCGTCATCCTTGTAATCTTGATGTGGCCTGTACTTCATG |  |
| *hopZ5-F* | CCCAAGCTTACAAGCCAGCGATAACACCTAC | For cloning *hopZ5* and *hopH1* together with *hopH1-R*; *Eco*RI site is underscored |
| pML-B3-F | TTAGAGCTGCTTAATGAGGTCGG | Detecting the vector pML-B3-free |
| pML-B3-R | CGGTAGATGATAGAGGTTGGGAT |  |
| 771-AcZLP1-F | ACGGGGGACGAGCTCGGTACCATGGCGATGATGGCAGTGAATGTGGTACTG | For coloing the AcZLP CDS carrying the homologous arms of nLUC. *Sal*I and *Kpn*I sites are underscored |
| 771-AcZLP1-R | CGCGTACGAGATCTGGTCGACACCCAAAACTTCGATTGTTTCAACATGT |  |
| 771-AeZLP1-F | ACGGGGGACGAGCTCGGTACCATGGCGATGATGGCAGTGAATGTGGTACTG | For coloing the AcZLP CDS carrying the homologous arms of nLUC. *Sal*I and *Kpn*I sites are underscored |
| 771-AeZLP1-R | CGCGTACGAGATCTGGTCGACACCCAAAACTTCGATTGTTTCAACATGT |  |
| 772-HopH1-F | TACGCGTCCCGGGGCGGTACCATGATCACTCCATCTCGATATCCA | For coloing the *hopH1* carrying the homologous arms of cLUC, *Sal*I and *Kpn*I sites are underscored |
| 772-HopH1-R | ACGAAAGCTCTGCAGGTCGACCTATTGATGTGGCCTGTACTTCATGC |  |
