# Supplementary figures and images for: Efficient genome editing in Pseudomonas syringae pv. actinidiae using the CRISPR/FnCas12a system
Source: Mol Hortic. 2025 Nov 3;5:60. doi: 10.1186/s43897-025-00180-0 (PMC12581509; doi:10.1186/s43897-025-00180-0)

Fig. S1

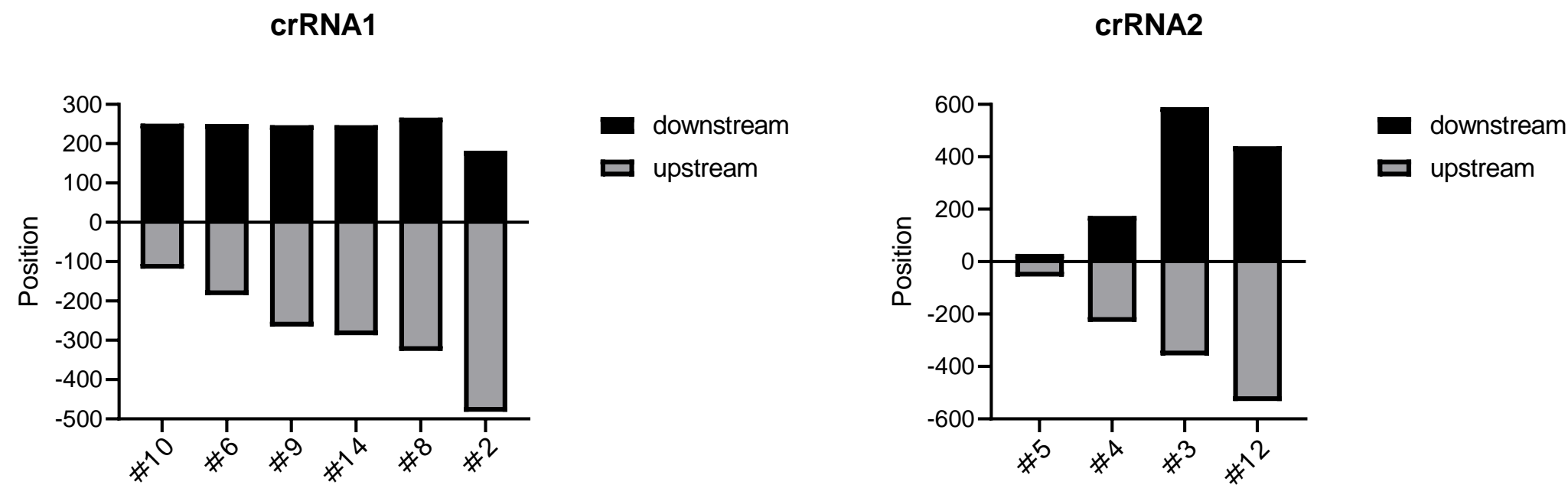

Supplement: Supplementary file 3 — Supplementary Material 3: Fig. S1. Location of hopH1 deletions generated by CRISPR/FnCas12a-mediated gene editing with crRNA1 and crRNA2. The first downstream base adjacent to the PAM is referred to as position 1, and the first upstream base adjacent to the PAM sequence is -1. The x-axes show the mutant designations, and the y-axes show the extent of deletion events. [file 43897_2025_180_MOESM3_ESM.pdf]

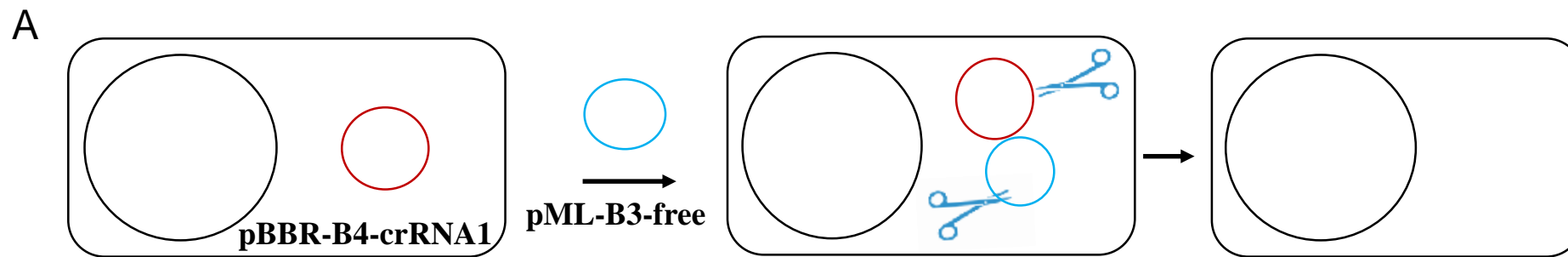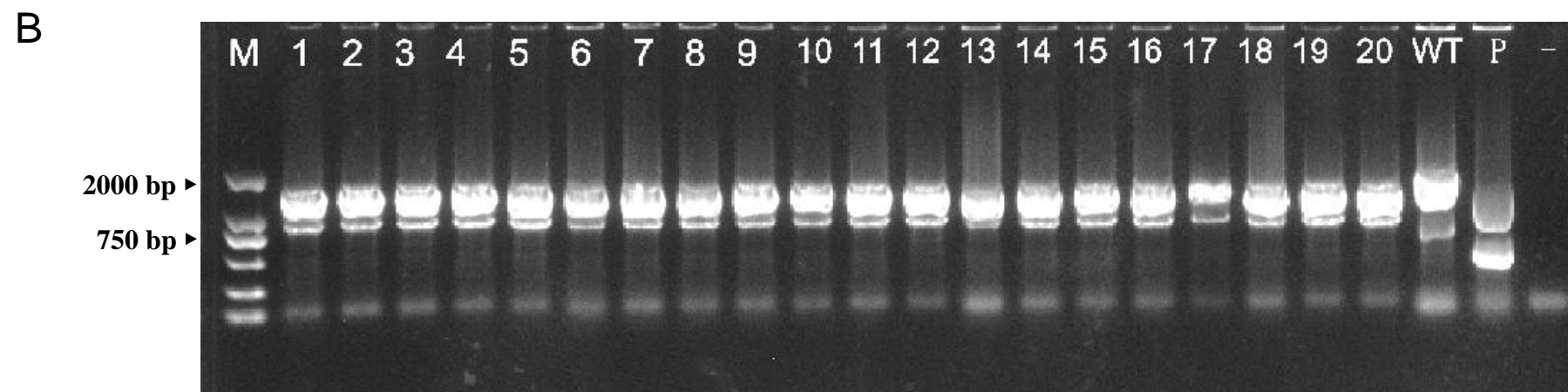

Supplement: Supplementary file 4 — Supplementary Material 4: Fig. S2. Plasmid curing and strain complementation. A Schematic showing how the FnCas12a nuclease-mediated cleavages of pML-B3-free that are mediated by oriV and LigD result in editing and removal of pBBR-B4-crRNA constructs. The red and blue circles represent pBBR-B4 and pML-B3, respectively. B Psa mutant #8 contains a 593-bp deletion in hopH1 and was selected for plasmid curing. pML-B3free was transferred into mutant #8 by conjugation, and colonies were then screened on LB plates with and without Km. Sensitivity to Km indicated that plasmid curing was successful, and this was verified by PCR. Individual Psa colonies (n = 16) were subjected to PCR with the primers RV-M/pBBR-B4-CHECK-R to detect whether pBBR-B4-crRNA (576 bp) was eliminated. Lanes: M, molecular weight marker; 1–20, single colonies randomly selected after plasmid curing; WT, wild-type Psa M228; P, pBBR-B4-crRNA1; -, ddH2O. [file 43897_2025_180_MOESM4_ESM.pdf]

**A**

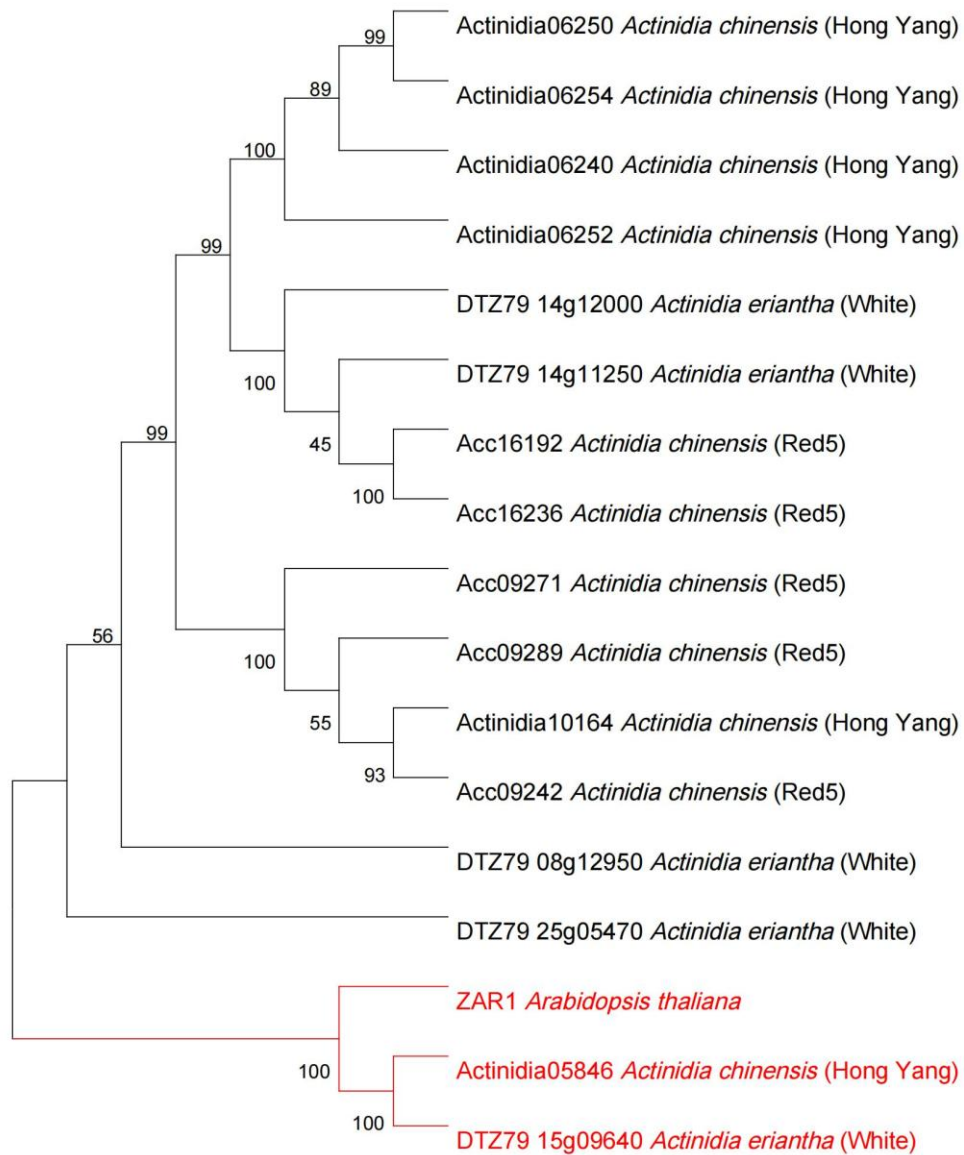

**B**

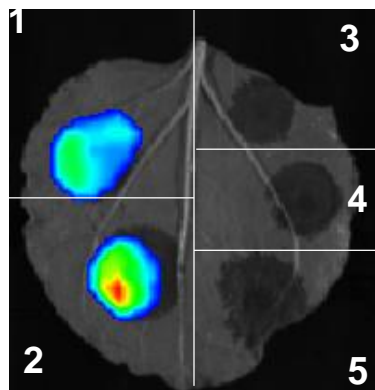

- 1: cLUC-HopH1+nLUC-AcZLP1
- 2: cLUC-HopH1+nLUC-AeZLP1
- 3: cLUC+nLUC-AcZLP1
- 4: cLUC+nLUC-AeZLP1
- 5: cLUC-HopH1+nLUC

Supplement: Supplementary file 5 — Supplementary Material 5: Fig. S3. HopH1 interacted with ZAR1 homologs from Actinidia species. A The phylogenetic tree of Arabidopsis ZAR1 in Actinidia species was construuted based on full length protein sequence by the maximum likehood method. B LCA showing that HopH1 interacted with the AeZLP1 and AcZLP1 in N. benthamiana. Empty vectors were used as negative controls. Four individual N. benthamiana plants were used in each replication, each treatment contained three replications and one similar image was used from three replications. [file 43897_2025_180_MOESM5_ESM.zip › Figure S3-PDF.pdf]
