# Supplementary material for: Efficient genome editing in Pseudomonas syringae pv. actinidiae using the CRISPR/FnCas12a system
Source: Mol Hortic. 2025 Nov 3;5:60. doi: 10.1186/s43897-025-00180-0 (PMC12581509; doi:10.1186/s43897-025-00180-0)
Supplement: Supplementary file 5 — Supplementary Material 5: Fig. S3. HopH1 interacted with ZAR1 homologs from Actinidia species. A The phylogenetic tree of Arabidopsis ZAR1 in Actinidia species was construuted based on full length protein sequence by the maximum likehood method. B LCA showing that HopH1 interacted with the AeZLP1 and AcZLP1 in N. benthamiana. Empty vectors were used as negative controls. Four individual N. benthamiana plants were used in each replication, each treatment contained three replications and one similar image was used from three replications. [file 43897_2025_180_MOESM5_ESM.zip › Figure S3.pptx]

## Slide 1
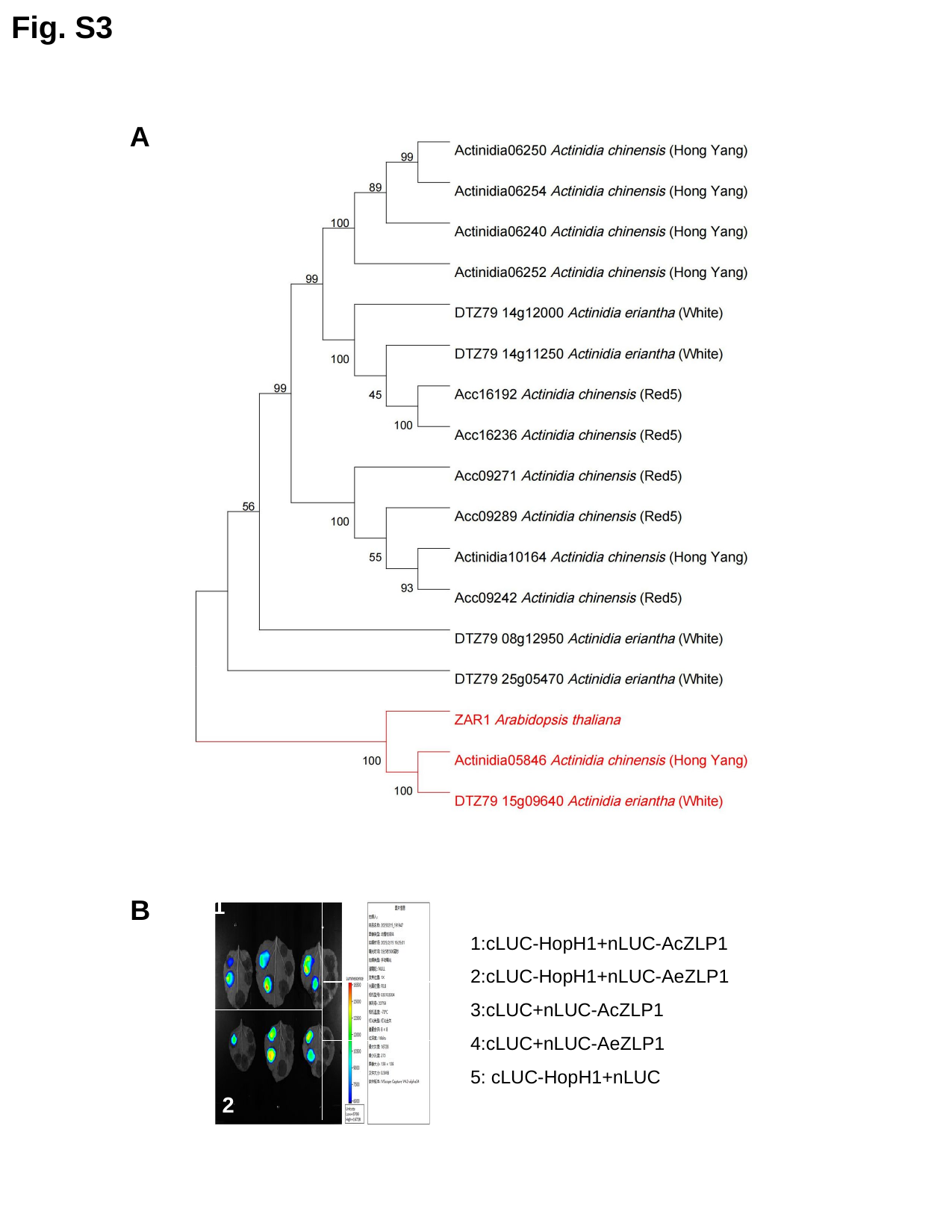

# Fig. S3
A
B
1
3
1:cLUC-HopH1+nLUC-AcZLP1
2:cLUC-HopH1+nLUC-AeZLP1
3:cLUC+nLUC-AcZLP1
4:cLUC+nLUC-AeZLP1
5: cLUC-HopH1+nLUC
4
2
5
